# The Use of Chitosan/Perlite Material for Microbial Support in Anaerobic Digestion of Food Waste

**DOI:** 10.3390/ma18153504

**Published:** 2025-07-26

**Authors:** Agnieszka A. Pilarska, Anna Marzec-Grządziel, Małgorzata Makowska, Alicja Kolasa-Więcek, Ranjitha Jambulingam, Tomasz Kałuża, Krzysztof Pilarski

**Affiliations:** 1 Department of Hydraulic and Sanitary Engineering, Poznań University of Life Sciences, Piątkowska 94A, 60-649 Poznan, Poland; malgorzata.makowska@up.poznan.pl (M.M.); tomasz.kaluza@up.poznan.pl (T.K.); 2 Department of Agriculture Microbiology, Institute of Soil Science and Plant Cultivation—State Research Institute, Czartoryskich 8, 24-100 Pulawy, Poland; agrzadziel@iung.pulawy.pl; 3 Institute of Environmental Engineering and Biotechnology, Faculty of Natural Sciences and Technology, University Opole, Kominka 6, 46-020 Opole, Poland; akolasa@uni.opole.pl; 4 CO_2_ Research and Green Technologies Centre, Vellore Institute of Technology, Vellore 632014, Tamil Nadu, India; ranjitha.j@vit.ac.in; 5 Department of Biosystems Engineering, Poznań University of Life Sciences, Wojska Polskiego 50, 60-627 Poznan, Poland; pilarski@up.poznan.pl

**Keywords:** chitosan/perlite system, physico-chemical properties, food waste, anaerobic digestion, process efficiency, NGS, FTIR

## Abstract

This study aims to evaluate the effect of adding a chitosan/perlite (Ch/P) carrier to anaerobic digestion (AD) on the efficiency and kinetics of the process, as well as the directional changes in the bacterial microbiome. A carrier with this composition was applied in the AD process for the first time. A laboratory experiment using wafer waste (WF) and cheese (CE) waste was conducted under mesophilic conditions. The analysis of physico-chemical properties confirmed the suitability of the tested carrier material for anaerobic digestion. Both components influenced the microstructural characteristics of the carrier: perlite contributed to the development of specific surface area, while chitosan determined the porosity of the system. Using next-generation sequencing (NGS), the study examined how the additive affected the genetic diversity of bacterial communities. Fourier-transform infrared spectroscopy (FTIR) revealed that the degradation rate depended on both the carrier and the substrate type. Consequently, the presence of the carrier led to an increase in the volume of biogas and methane produced. The volume of methane for the wafer waste (WF–control) increased from 351.72 m^3^ Mg^−1^ (VS) to 410.74 m^3^ Mg^−1^ (VS), while for the cosubstrate sample (wafer and cheese, WFC–control), it increased from 476.84 m^3^ Mg^−1^ (VS) to 588.55 m^3^ Mg^−1^ (VS).

## 1. Introduction

Anaerobic digestion is a complex process consisting of a number of microbial transformations, whereby organic substrates are converted into biogas containing mainly methane and carbon dioxide [1]. Anaerobic decomposition of organic matter is presently applied in the treatment of wastewater, sludge from wastewater treatment plants, and slurry. It is also increasingly being used to generate energy from various types of agri-food waste, which is gradually displacing plant substrates from purpose-grown crops [2]. The microbial community within an anaerobic reactor consists of three main groups: fermentative bacteria, acetic acid bacteria, and methanogenic archaea. Among these, methanogenic archaea are the most susceptible to environmental changes, as their growth rate is slower compared to fermentative and acetic bacteria [3].

Considering the critical role of methanogenic archaea density and diversity in anaerobic digestion, considerable effort is focused on establishing and maintaining a stable and active population of these microorganisms [4]. To this end, microbial carriers are used in biotechnological processes, including AD. Bacteria and archaea involved in methanogenesis form biofilms by adhering to or attaching to the carrier material. Properly selected biofilm carriers prevent the leaching of microorganisms from sludge, ensure a high density of methanogens, and thus contribute to highly efficient methane production. Therefore, biofilm carriers in anaerobic digestion reactors have the potential to enhance reactor productivity. An optimal cell carrier ought to possess a highly developed specific surface area, adequate porosity, biocompatibility, stability, low density, and sufficient mechanical strength [5]. Additionally, the support matrix should be cost-effective in practical applications (e.g., as a waste material) and non-toxic to both the immobilised microorganisms and the environment.

To date, various types of biofilm carriers have been developed for AD, including stand-alone materials and integrated systems, which perform specific functions and have both advantages and certain disadvantages (e.g., small pore diameters, dissolution, low efficiency, high cost, and limited availability). Immobilising microbial cells on various zeolite structures has demonstrated beneficial effects [6]. In general, zeolites are most commonly used as carriers in AD due to their favourable characteristics for microorganism adhesion and their ability to remove ammonium ions. The promotion of surface contact and the improvement of interspecies electron transport (when using conductive materials such as magnetite) have had a significant impact on treatment results [7,8]. Modern spectroscopy techniques and next-generation sequencing provide information on biofilm composition and structural organisation, elucidating the function of the additive [9].

Developing technologies using microbial carriers in anaerobic degradation is therefore necessary and justified. The authors, drawing on their experience [5,8,9], have developed a new system to support AD based on promising materials: chitosan and perlite. Chitosan, a naturally occurring polysaccharide, is typically derived from chitin extracted from the shells of marine invertebrates. This material has several valuable physicochemical properties, such as biocompatibility, non-toxicity, the ability to form polycations in acidic media, and ease of modification [10]. It is also known for its bioactive properties, including its ability to promote microbial adhesion, chelate metal ions, and adsorb organic compounds. These characteristics result from the presence of functional amino and hydroxyl groups, which act as chemically active centres enabling diverse biochemical interactions [11,12]. Additionally, chitosan exhibits antimicrobial activity, which can help selectively enhance the growth of beneficial microbial communities [13]. These characteristics make it applicable in wastewater treatment, anaerobic digestion, and various separation processes, as well as in medicine and pharmaceuticals [14]. It is worth noting that, in comparison with conventional microporous materials employed in separation processes, nanochitosan exhibits superior functional characteristics, mainly due to its increased specific surface area and enhanced adsorption capacity [15]. The literature reports highlight the pronounced biocatalytic influence of chitosan, as well as chitosan–magnetite and chitosan–titania–magnetite composites, on enhancing biogas yield and methanogenic activity [16,17,18]. However, despite these advantages, chitosan alone has limitations in terms of mechanical strength and stability under the operational conditions often encountered in industrial processes.

Perlite, on the other hand, is an igneous rock composed of acidic rhyolitic volcanic glass with excellent structural stability [19]. Its high surface area and porosity create an ideal environment for microbial colonisation, enhancing the immobilisation of microorganisms and facilitating the efficient mass transfer of nutrients and gases. These properties have contributed to the use of perlite as a metal adsorbent [20,21]. Perlite is also chemically inert, non-toxic, insoluble in water, and inexpensive. Therefore, chitosan has been investigated and applied as a microbial carrier in the construction industry to enhance the durability of concrete structures [22].

Regarding mineral carriers in anaerobic digesters, it should be noted that many types have been investigated to date, including bentonite, sepiolite, talc, vermiculite, montmorillonite, and diatomite. Some of these have been found to facilitate start-up, while others contribute to chemical oxygen demand (COD) removal. Perlite has so far been rarely used; however, Ivankovic et al. (2022) demonstrated that it can be successfully used as a support material in anaerobic digestion [23].

Combining chitosan with perlite into a hybrid system is thus grounded in the concept of a synergistic effect arising from the complementary physicochemical properties and functionalities of the two materials. The chitosan–perlite interaction could enhance the material’s surface area and functionality by utilising the mechanical stability and structural support of perlite, while the chitosan coating introduces active binding sites and bioactivity. This combination shows great potential for developing a durable, effective, and versatile system applicable across various biotechnological and environmental fields, such as anaerobic biodegradation. In addition to performance benefits, the hybrid system offers significant practical advantages. Chitosan is relatively expensive, so incorporating perlite reduces material costs while maintaining high efficiency. Both materials are environmentally friendly and align with green chemistry principles, supporting the transition towards sustainable industrial practices.

It is also worth noting that research on the synthesis and performance of the chitosan/perlite composite, as well as other materials based on this system, has been conducted for over a decade. Its main application is the adsorption of polluting compounds, including pigments [24,25]. Based on kinetic and thermodynamic studies, chitosan-coated expanded perlite has been shown to be an effective adsorbent. Additionally, this material can serve as a matrix for catalysts in technological processes [26]. However, as a system of these two materials, it has not yet been tested in anaerobic digestion for any of the functions mentioned.

This study aims to evaluate the impact of a microbial support not previously applied in the AD process, composed of a chitosan/perlite system at a 3:1 mass ratio, on the stability and efficiency of food waste biodegradation in a batch reactor under mesophilic conditions. The assessment also covers changes in microbial diversity under the influence of the added carrier (in-process), qualitative changes in the molecular structure of the decomposed materials and quantitative changes using next-generation sequencing (NGS), FTIR, and the classic drying–weighing method, respectively. This paper also presents the analyses of the most relevant physico-chemical parameters of the studied carrier. It should be emphasised that this study aims to investigate the synergistic effects between chitosan and perlite, focusing on how their complementary properties contribute to enhanced system performance. This article is a continuation of the authors’ research on materials used as microbiological carriers.

## 2. Materials and Methods

### 2.1. Feedstocks and Additional Materials

In the studies, selected confectionery waste—waste wafers (WF)—were used as substrates, both on their own and in combination with cheese waste as a cosubstrate (WFC). Both feedstocks were sourced from manufacturers near Poznań (Wielkopolskie Voivodship). On the other hand, digested sewage sludge (SS) used as inoculum—as in other experiments by the authors—was obtained from the Poznań municipal sewage treatment plant. The benefits of conducting the AD process in a digested SS environment are scientifically proven—the buffering and stabilising effects of this material have been described in numerous studies [8,27]. The basic physico-chemical parameters of these materials are presented in Table 1.

**Figure 1:**
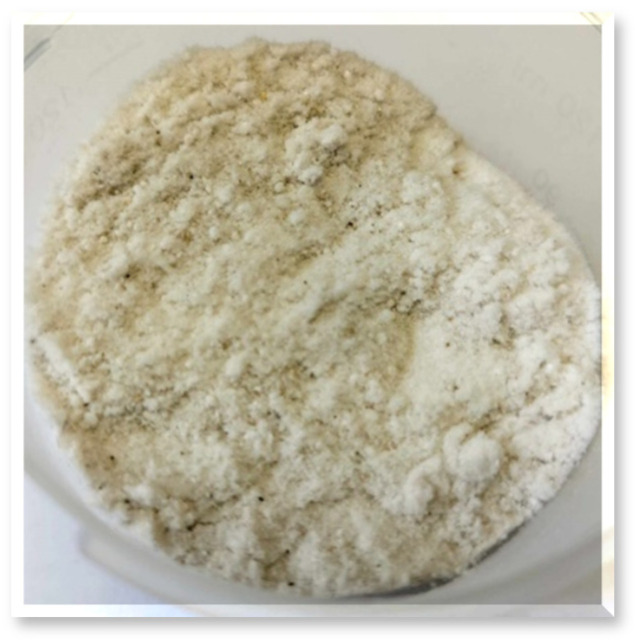
Microbial carrier consisting of a Ch/P (3:1) system.

Additional materials used as microbial carriers in AD included chitosan from crab shells (degree of deacetylation: 70%) from Sigma–Aldrich, Saint Louis, MO, USA, and perlite, which is an amorphous mineral consisting of fused potassium aluminium sodium silicate (*Biomus* sp. z o.o., Lublin, Poland). Both materials were purchased as powder and combined in a weight ratio 3:1 (Ch/P), see Figure 1.

The physico-chemical properties of the constituent materials of the carrier and the system are detailed in the Results and Discussion.

Both substances, chitosan and perlite, were placed in the bioreactor in the amount of 20 g per 1 L of feedstock, following the positive effects observed in previous studies [27]. The hybrid system was produced by simultaneously grinding and mixing the constituent components using a ball mill (PULVERISETTE 23, FRITSCH GmbH–Milling and Sizing, Idar-Oberstein, Germany). The resulting material was subsequently rinsed with phosphate-buffered saline (PBS), treated with sterile distilled water, and dried at 70 °C until a constant initial weight was achieved. In general terms, the ratio of materials in the developed carrier system was established based on studied and compared physico-chemical properties.

The specific factors considered in determining the quantitative ratio of components in a hybrid Ch/P material are as follows: (i) the intended application, (ii) the properties of both materials, (iii) their cost and availability, and (iv) the interactions between the two components. The optimal chitosan-to-perlite ratio in the hybrid system depends on the specific application (e.g., adsorption, catalysis, bioremediation, or use as a carrier in biotechnological processes) and the desired material properties. Given the physicochemical properties of both materials, it should be acknowledged that a higher chitosan content enhances the availability of functional groups while maintaining high bioactivity and adsorption capacity. Conversely, a higher perlite content ensures mechanical integrity, improves dispersion, and prevents potential aggregation of chitosan particles. Since the material used in the present study was intended as a cell carrier, a significant excess of chitosan (constituting 75% of the composition) was selected to facilitate the immobilisation/adsorption of microorganisms in the AD process, which occurs in a sewage sludge environment serving as an inoculum. The literature data confirm that the most effective adsorption of chemical compounds onto chitosan/perlite material occurs at a pH close to neutral, which aligns with the conditions of anaerobic degradation [24]. This indicates that the chitosan/perlite system in a 3:1 ratio primarily functions to support enzymes and cells by stably binding them via chemical interactions on the surface. Additionally, it may act as a biosorbent for chemical compounds that could potentially inhibit the process. Although this latter function was not investigated in the present study, it is a well-documented property of chitosan [12]. Trial experiments and theoretical considerations indicated that a 3:1 ratio provides an optimal balance between adsorption capacity and the mechanical properties required for this process. This approach ensures that the material retains its dual functionality as both a carrier and a biosorbent. In the case of conducting the AD process in a less toxic environment (other than sewage sludge), a chitosan content of 60–65% is likely to be sufficient [28].

As the present research is pioneering and dedicated to a specific carrier composition, further studies are recommended to optimise the chitosan-to-perlite ratio in relation to system efficiency.

### 2.2. Physico-Chemical Analysis of Substrates and Carriers

#### 2.2.1. Feedstock, Inoculum, and Batch Analysis

The pH (potentiometric method) and electrolytic conductivity of the feedstocks, inoculum, and digestate samples were measured using an Elmetron CP–215 apparatus (Elmetron R., R. i A. Olszewscy Sp. j., Zabrze, Poland). Total solids (TS) content was determined by drying the samples at 105 °C using a Zalmed SML dryer (Zalmed, Łomianki, Poland), while volatile solids (VS) were quantified through combustion at 550 °C in an MS Spectrum PAF 110/6 furnace (MS Spectrum, Warsaw, Poland), following gravimetric analysis procedures.

Carbon content was established by combustion at 900 °C, followed by CO_2_ determination via infrared spectrometry using an OI Analytical Aurora 1030W TOC Analyzer (Picarro Inc., Santa Clara, CA, USA). Nitrogen content was assessed through the Kjeldahl method involving digestion and titration with 0.1 M HCl using Tashiro’s indicator (Chempur, Piekary Slaskie, Poland). Ammonium nitrogen was determined by distillation followed by titration with 0.1 M HCl, also in the presence of Tashiro’s indicator. Phosphorus content was analysed after mineralisation in nitric acid using an UltraWAVE microwave digestion system (Milestone Inc., Fremont, CA, USA), followed by spectrophotometric measurement with a Varian Cary 50 spectrophotometer (Varian Inc., Palo Alto, CA, USA).

#### 2.2.2. Analysis of Carrier Materials

Carrier materials, individually and in a Ch/P (3:1) binary system, were described in terms of physico-chemical and microstructural properties.

In the initial stage of the analysis, particle size distribution of the materials was determined using a Zetasizer Nano ZS instrument (Malvern Instruments Ltd., Worcestershire, UK), equipped with a 4 mW helium–neon laser. The system operates based on non-invasive backscattering (NIBS), allowing for the measurement of particles ranging from 0.6 to 6000 nm. Prior to measurement, the samples were dispersed in isopropanol and subjected to mild sonication to ensure uniform suspension. All measurements were conducted at a constant temperature of 25 °C. The particle size distribution was characterised using cumulant analysis, which yields a width parameter expressed as the polydispersity index (PdI).

The morphology and microstructure of chitosan and perlite were investigated using scanning electron microscopy (SEM). Micrographs were obtained with a FEI Quanta FEG 250 microscope (FEI Company, Hillsboro, OR, USA), operating in low vacuum mode (70 Pa) at an accelerating voltage of 10 kV. Prior to imaging, samples were coated with a thin layer of gold for 5 s using a Balzers PV205P sputter coater (Oerlikon Balzers, Balzers, Liechtenstein). Elemental composition was determined by energy-dispersive X-ray spectroscopy (EDS), performed at an accelerating voltage of 10 kV using an Octane SDD detector (EDAX Inc., Mahwah, NJ, USA). The contents of carbon, oxygen, and silicon were identified.

The chemical composition of the materials was further analysed using a FLASH 2000 elemental analyser (Thermo Fisher Scientific, Waltham, MA, USA), employing a dynamic combustion method. Approximately 2–4 mg of each sample was weighed into tin capsules and introduced into the combustion reactor via an autosampler, with oxygen supplied in a controlled amount. Combustion was carried out at 900–1000 °C, and the resultant gases were transported by a helium stream through a copper-filled secondary furnace, then to a chromatographic column via a water trap for product separation. Detection was performed using a thermal conductivity detector (TCD), and results were processed with Thermo Scientific Eager Xperience software (Thermo Fisher Scientific, Waltham, MA, USA; available at: https://www.thermofisher.com; accessed: 25 July 2025).For oxygen analysis, the instrument was operated in pyrolysis mode. Samples were weighed in silver capsules and introduced into a reactor containing nickel-coated carbon at 1060 °C. Oxygen was released as carbon monoxide, which was separated chromatographically and detected by TCD.

The porous structure of the samples was characterised by nitrogen adsorption/desorption isotherms at 77 K, recorded using an ASAP 2420 analyser (Micromeritics Instrument Co., Norcross, GA, USA). Samples were degassed at 90 °C under vacuum prior to analysis. Specific surface area (SSA) was calculated using the Brunauer–Emmett–Teller (BET) model over the relative pressure range 0.05 < P/P_0_ < 0.25, while the mean pore size and total pore volume were determined using the Barrett–Joyner–Halenda (BJH) method.

The functional groups of the studied powders were identified by Fourier-transform infrared spectroscopy (FTIR), using a Vertex 70 spectrophotometer (Bruker, Bremen, Germany) at room temperature. Samples were analysed as KBr pellets, prepared by pressing a mixture of approximately 0.25 g of anhydrous KBr and 1 mg of sample in a stainless-steel ring at a pressure of 10 MPa. Spectra were recorded in transmission mode over the range of 4000–400 cm^−1^ with a resolution of 0.5 cm^−1^.

Thermal behaviour of the samples was investigated by differential scanning calorimetry (DSC) using a Netzsch DSC 204 F1 Phoenix instrument (Netzsch Analyzing & Testing, Selb, Germany). Each 5 mg sample was heated from 25 °C to 500 °C at a rate of 10 °C/min under a nitrogen atmosphere (10 mL/min) and held isothermally at 500 °C for 5 min. Subsequently, the samples were cooled to 20 °C under the same nitrogen flow.

### 2.3. Experimental Setup for Biogas Generation

#### 2.3.1. Sample Preparation Procedure

Control samples prepared using only waste food (WF) and a system of waste food with cosubstrates (WFC) were tested under the following designations: WF–control, WFC–control, and—where the Ch/P carrier was applied—WF–Ch/P and WFC–Ch/P, respectively.

As noted earlier, the primary objective of this study was to assess the effect of the hybrid system formed by the combination of both carrier components. Using samples containing only food waste as controls, a comparative analysis was conducted to assess microbiological and chemical changes occurring throughout the process as a result of the addition of Ch/P. As stated, both individual confectionery waste and a cosubstrate system (a combination of confectionery and dairy waste) were tested, primarily for practical and economic reasons. The rationale for conducting tests focused exclusively on the combined system of both components is based on well-established theoretical knowledge, as well as previous research on their application, both individually and in combination [15,16,23,24,25,26]. Fundamentally, the authors of this study base their research concept on the following grounds: (i) both components rely on each other for functionality—perlite serves as a structural matrix for immobilisation, while chitosan acts as a bioactive substance for adsorption—meaning that their individual effects may not be meaningful or biologically relevant; (ii) a key focus of the present study is to examine the synergistic effects of the two combined components, as previous tests of the system in other applications have demonstrated exclusively positive effects; and (iii) prior research confirms that the components do not exhibit notable functionality individually, and the number of studies investigating various systems using these components, either separately or in combination, strongly supports shifting the focus directly to the hybrid system [16,23,24,25,26,28].

The composition of the batches was determined based on the German standard VDI 4630 [29], according to which the dry matter content of the mixtures did not exceed 10%. Table 2 summarises the batch compositions along with key physico-chemical characteristics of the substrates and inoculum relevant to the AD process.

The uncertainty value was determined based on repeated measurements performed under identical conditions for each of the analysed variants, while also taking into account the accuracy of the equipment used (including laboratory reactors and the gas analyser).

It should be noted that statistical analysis of the measurement results—particularly the evaluation of the standard deviation and Type A uncertainty—constitutes an integral component of the estimation of total measurement uncertainty. Type A uncertainty is based on empirical data obtained from repeated measurements, with its value calculated from the statistical dispersion of the results [30]. Therefore, the application of statistical analysis (arithmetic mean, standard deviation, mean uncertainty, and confidence level) directly contributes to the quantitative assessment of uncertainty. In the context of the present study, statistical evaluation of the results (e.g., biogas yield) enabled the determination of the range within which the repeatability of measurements can be expected, which corresponds to one of the components of total measurement uncertainty.

In the presentation of empirical data involving chemical analyses, statistical (Type A) uncertainty represents only a part of the overall uncertainty. The second component—Type B uncertainty—originates, for instance, from the measuring equipment. Consequently, both components were combined using the root-sum-of-squares method, and the final value of uncertainty was expressed numerically, with reference to the confidence level.

In order to quantitatively assess the uncertainty of the results related to biogas yield, an analysis was conducted of the identified sources of error, such as measurement repeatability, the accuracy of measuring equipment, and variability in sample preparation. The combined standard uncertainty, *u_c_*, was estimated using the error propagation method, assuming the independence of individual components [30]:

(1) $$u_{c}=\sqrt{u_{1}^{2}+u_{2}^{2}+\cdots u_{n}^{2}}$$

The expanded uncertainty, *U*, was calculated using a coverage factor *k* = 2, which corresponds to a confidence level of 95%:

(2) $$U=k\cdot u_{c}$$

#### 2.3.2. Anaerobic Digestion Procedure

**Figure 2:**
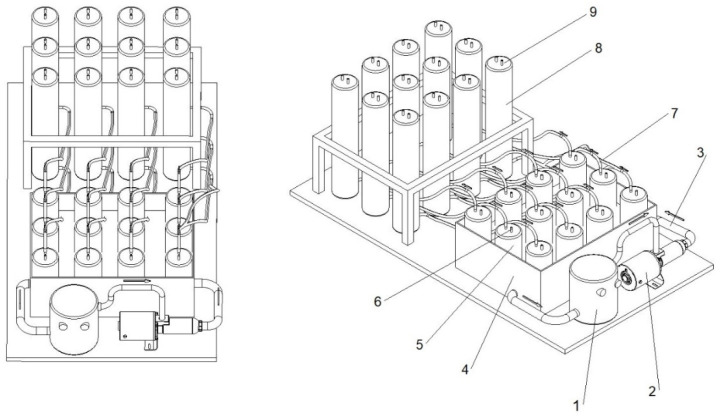
The anaerobic bioreactor (with a 12-chamber section) used to produce biogas in the experiment: 1—temperature-controlled water reservoir; 2—circulation pump; 3—thermally insulated tubing; 4—external water jacket maintained at 39 °C; 5—anaerobic reactor vessel (1.4 L); 6—valve for digestate collection; 7—gas outlet conduit; 8—calibrated gas collection cylinder; 9—port for gas sampling (adapted from [27]).

Anaerobic digestion experiments were conducted using a multi-chamber bioreactor system, as illustrated in Figure 2. The setup consisted of 12 individual reactors, allowing each of the 4 experimental variants to be tested in triplicate.

The substrates were introduced into 1.0 L digestion chambers (5) and manually stirred once daily. The digesters were placed within a water-filled vessel (4) linked to a thermal control unit (1), which ensured stable mesophilic temperature conditions throughout the process. The biogas generated during anaerobic digestion was channelled (7) into calibrated collection cylinders (8), where it was quantitatively captured and stored, following the setup presented in [27].

In accordance with the German DIN 38 414–S8 standard [31], the digestion process was continued until the daily biogas production declined to less than 1% of the cumulative yield. The hydraulic retention time (HRT) for the experiment was 20 days. Biogas volumes were recorded every 24 h.

The composition of the produced biogas—including methane (CH_4_), carbon dioxide (CO_2_), hydrogen sulphide (H_2_S), ammonia (NH_3_), and oxygen (O_2_)—was determined using a GA5000 portable gas analyser (Geotech, Coventry, UK). For verification and supplementary analysis, Mg–72 and Mg–73 sensors (Alter Inc., Tarnowo Podgórne, Poland) were employed. The detection ranges for the gases were as follows: CH_4_ (0–100%), CO_2_ (0–100%), O_2_ (0–25%), H_2_S (0–2000 ppm), and NH_3_ (0–1000 ppm). Experimental results were used to determine the biogas yield per unit of total and volatile solids, expressed in m^3^ Mg^−1^.

#### 2.3.3. Physico-Chemical Analysis of Samples

In addition to monitoring the pH of fermentation slurry samples collected at various stages of the process (according to the procedure outlined in Section 2.2), the concentrations of volatile fatty acids (VFA), total alkalinity (TA), and the resulting VFA/TA ratio were determined. For this purpose, 5 mL of each sample was subjected to titration with a 0.1 N sulphuric acid (H_2_SO_4_) solution. The TA was quantified by titrating to pH 5.0, while the VFA content was subsequently assessed through a second titration step, lowering the pH from 5.0 to 4.4.

The samples were additionally subjected to FTIR to visualise changes in their chemical structure and to obtain information on the dynamics of organic matter decomposition during AD (methodology as in Section 2.2.2). The collected samples were continuously prepared for analysis by drying in a stationary laboratory dryer, SLN 75 (POL–EKO sp.k., Wodzisław Śląski, Poland), at 105 °C for 48 h until a powder was obtained. After weight analyses and FTIR, the samples were calcined in an NT 1313 laboratory furnace (Neoterm, Wrocław, Poland) at 550 °C for 2 h to determine the mass of organic matter.

#### 2.3.4. Microbiological Analysis of Samples

In order to investigate and compare qualitative and quantitative changes occurring in the anaerobic bioreactor, control and Ch/P carrier samples were analysed using NGS, as described in previous papers [9,32]. The material collected from the bioreactors during the first and last phases of the process was designated as follows: WF–Control 1, WF–Control 2, WF–Ch/P 1, WF–Ch/P 2, WFC–Control 1, WFC–Control, WFC–Ch/P 1, and WFC–Ch/P 2.

Genomic DNA was isolated from 500 mg of each sample using the Genomic Mini AX Soil kit (A&A Biotechnology, Gdynia, Poland), in accordance with the manufacturer’s protocol. Quantification of the extracted DNA was carried out with the Quant-iT High Sensitivity dsDNA assay kit (Invitrogen, Carlsbad, CA, USA), and measurements were performed using a Qubit2 fluorometer (Invitrogen). Additionally, 2 µL of each extract was assessed by electrophoresis on a 0.8% agarose gel to verify DNA quality.

The metagenomic workflow targeted the V3–V4 hypervariable region of the bacterial 16S rRNA gene. Amplification and library construction were performed using the 341F and 785R primers. Polymerase chain reactions (PCR) were conducted with the Q5 Hot Start High-Fidelity DNA Polymerase Kit (NEB Inc., Ipswich, MA, USA), adhering to the thermal cycling parameters recommended by the manufacturer.

Sequencing was executed using the Illumina MiSeq platform, applying 2 × 250 bp paired-end (PE) technology and the Illumina v2 chemistry kit, as described in reference [9]. Amplification followed the Illumina 16S Metagenomic Sequencing Library Preparation protocol (V3–V4), provided by Illumina, Inc. (San Diego, CA, USA). Sequencing data were acquired using the Illumina MiSeq PE300 system and analysed via the BaseSpace platform (Illumina, Inc., San Diego, CA, USA), with laboratory procedures and data processing carried out by Genomed S.A. (Warsaw, Poland). Automated analyses employed the 16S Metagenomics pipeline (version 1.0.1).

All downstream bioinformatics procedures were conducted in R environment (version 3.6.0) [33], using the DADA2 package (version 1.14) [34]. This included error model learning (learnErrors), identification of exact amplicon sequence variants (dada), and elimination of chimaeras (removeBimeraDenovo). Taxonomic classification was performed using the IDTAXA algorithm [35] in conjunction with the Ribosomal Database Project (RDP) database, version 18 [36]. The resulting datasets were managed and visualised using the phyloseq package (version 1.22.3) [37].

## 3. Results and Discussion

### 3.1. Physico-Chemical Properties of Carrier Materials

#### 3.1.1. Morphological and Microstructural Properties

**Figure 3:**
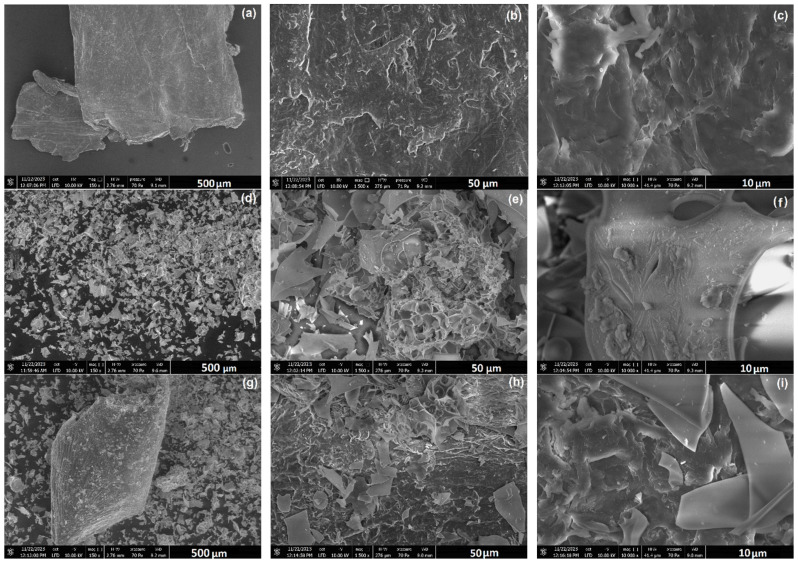
SEM images of chitosan (**a**–**c**); perlite (**d**–**f**); and chitosan/perlite (**g**–**i**) at various magnifications.

SEM images at various magnifications (see Figure 3a–i) demonstrate the morphological and microstructural characteristics of chitosan (Figure 3a–c) and perlite (Figure 3d–f) as components of the carrier material in Figure 3g–i. Images of pure chitosan show a heterogeneous surface with numerous grooves and indentations (Figure 3b,c), typical of crustacean carapaces [38]. Microscopic images of perlite show a porous, spongy (Figure 3e) microstructure formed by the passage of this material from liquid magma (during volcanic eruptions) into seawater [21]. In water, the perlite solidifies instantly, which causes the vapour bubbles within it to close, hence the holes and cracks in the images (Figure 3f).

The result of combining the two materials at a 3:1 ratio was a highly heterogeneous, in places, rough microstructure with discernible aggregates and agglomerates of particles [39]. The formed niches provide an ideal point for cell immobilisation. The specific and functional microstructure of the Ch/P system has previously been applied as a biosorbent for impurity removal and as an efficient catalyst for the rapid decolourisation of azo dyes [40]. The formation of cross-linked Ch/P composites increased the sorption capacity of chitosan.

The average chitosan particle diameters measured with the Zetasizer were 2605 nm, with 48.1% of 957.1 nm particles. The high PdI of 0.892 indicates significant particle agglomerates in the sample. These results confirm a highly heterogeneous powder microstructure with micrometre-sized particles. The results are similar for perlite, with the Z–average at 1242 nm (particles with diameters of 508.7 nm cover 56.3% of the sample) and PdI = 0.655. The sample of the chitosan/perlite system has the tendency to form particles of significant size (Z–average 1785 nm) and high PdI (0.855). These results correlate with the literature data, where micrometre-sized powders were used in the studies [41]. Nguyen et al. (2017) [42] observed the dependence of chitosan particle size and size distribution on zeta potential. They found that larger particle diameters could result in a decrease in zeta potential, implying lower dispersion stability [42].

#### 3.1.2. BET Surface Area and Pore Structures

**Figure 4:**
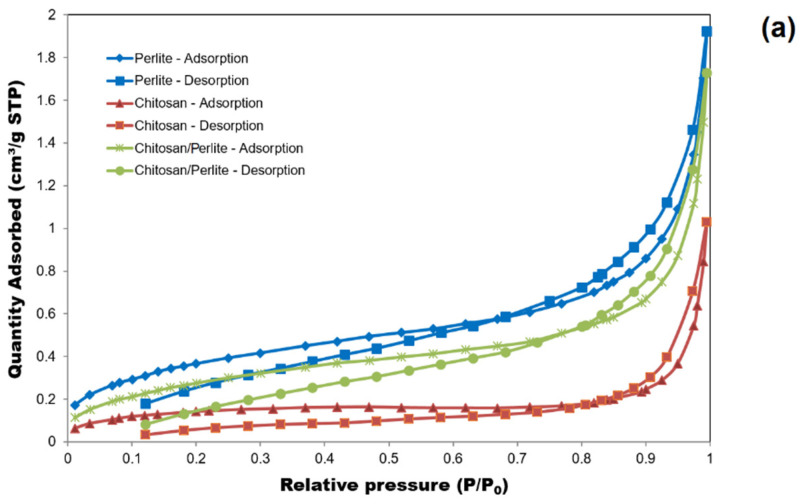

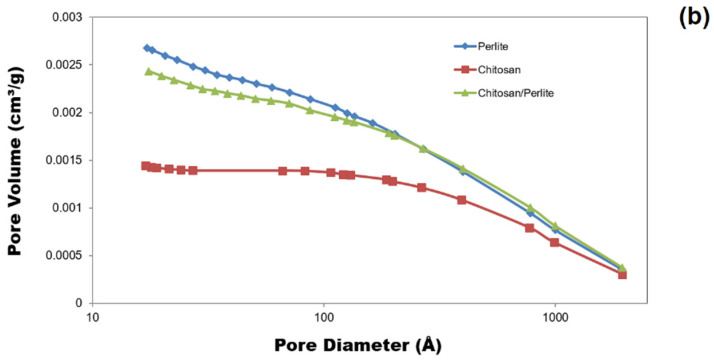
Nitrogen adsorption/desorption isotherms (**a**) and corresponding pore size distribution profiles (**b**) for chitosan, perlite, and the chitosan/perlite samples.

The microstructure of materials directly affects the development of the specific surface area, which is a key parameter for cell carriers. Figure 4a,b presents the nitrogen adsorption/desorption isotherms for the chitosan, perlite, and chitosan/perlite system materials. The shape of the isotherms is indicative of a mesoporous structure. A slight increase in nitrogen uptake was observed in the materials up to a relative pressure (p/p_0_) of 0.8. Above this value, as indicated by the shape of the isotherms, the amount of nitrogen adsorbed by the materials increased rapidly, reaching the highest value for perlite, followed by the chitosan/perlite carrier, and the lowest for chitosan. The spongy and porous structure of perlite proved conducive (see Figure 4b), which, together with the smaller particle size (compared to chitosan), promoted the development of the specific surface area. Smaller particle diameters of the material develop the specific surface area (especially the nanoparticles) to a large extent and shape their specific and unique properties. As indicated in Table 3, perlite exhibited the greatest BET-specific surface area (1.3730 m^2^/g) as well as the highest total pore volume (0.002678 cm^3^/g).

Given that the BET surface area of the chitosan sample was the smallest (0.5061 m^2^/g), it suggests that perlite was decisive for the development of the microstructural properties of the carrier, in particular, its specific surface area (despite the three times lower proportion of this component in the sample). However, the considerable chitosan pore diameters (up to 27,366 nm) are important for the immobilisation of microorganisms. The use of a combination of both materials in numerous ‘green’ applications, including as a biosorbent, is therefore justified.

The results obtained for the chitosan/perlite carrier represent a favourable superposition of the properties of both materials. The resulting material exhibits a well-developed surface area, comparable in size to perlite, with substantial pore diameters (Table 3). This confirms the appropriate selection of the Ch/P component ratio.

Potentially, the use of a lower chitosan content (i.e., less than 75%) could, as suggested by the diagram in Figure 4b, contribute to maintaining a larger pore volume. As previously mentioned in this study, it is advisable to further test carriers with a chitosan content of 60–65%. Nevertheless, a high proportion of chitosan, as confirmed by the literature data, promotes the formation of pores with significant average diameters, which undoubtedly facilitates cell immobilisation [28].

#### 3.1.3. Elemental Analysis and FTIR Spectra

**Figure 5:**
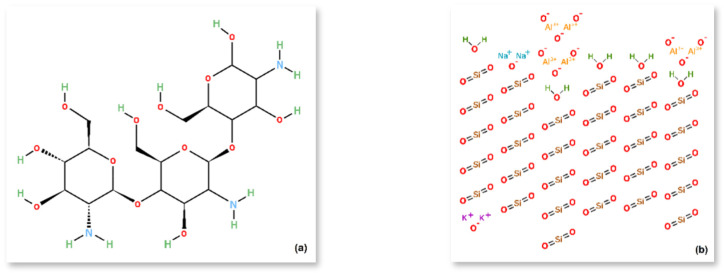
Structural formula of (**a**) chitosan and (**b**) perlite (authors’ scheme).

Chitosan is chemically defined as a copolymer of 2-acetamido-2-deoxy-n-β-d-glucopyranose and 2-amino-2-deoxy-β-glucopyranose (see Figure 5a). The sorption properties of this material towards metal ions, cholesterol, and proteins are due to the reactive amine and hydroxyl groups in its macromolecule. Perlite, on the other hand, is a rock of volcanic origin, containing only a small percentage of water. Its chemical composition is determined by the proportion of numerous oxides, with silica SiO_2_ (72%) and Al_2_O_3_ (14%) dominating, and the remainder formed by metal oxides, including K_2_O (4%), Na_2_O (3%), Fe_2_O_3_ (1%), CaO (1%), and MgO (0.5%). The structural formula of perlite is presented in Figure 5b.

Table 4 provides the chemical composition of chitosan, perlite, and the hybrid carrier material. The elemental analysis confirms that chitosan comprises four elements: nitrogen and aluminium, and the dominant carbon and oxygen. In the case of perlite, and consequently the system of the two analysed types of material, the chemical composition is enriched with other elements, both non-metals and metals (see Table 4).

**Figure 6:**
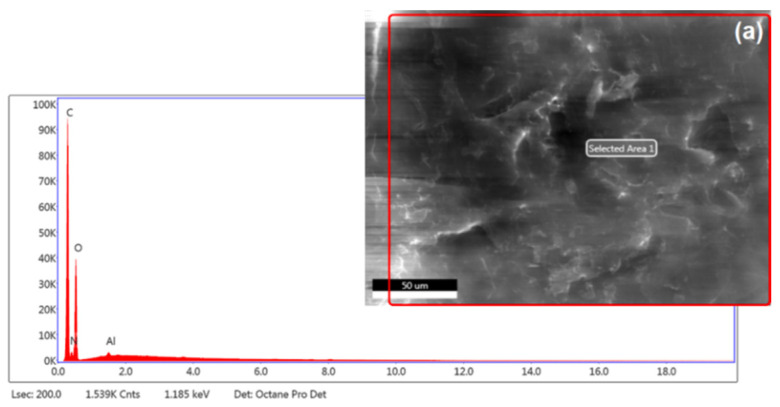

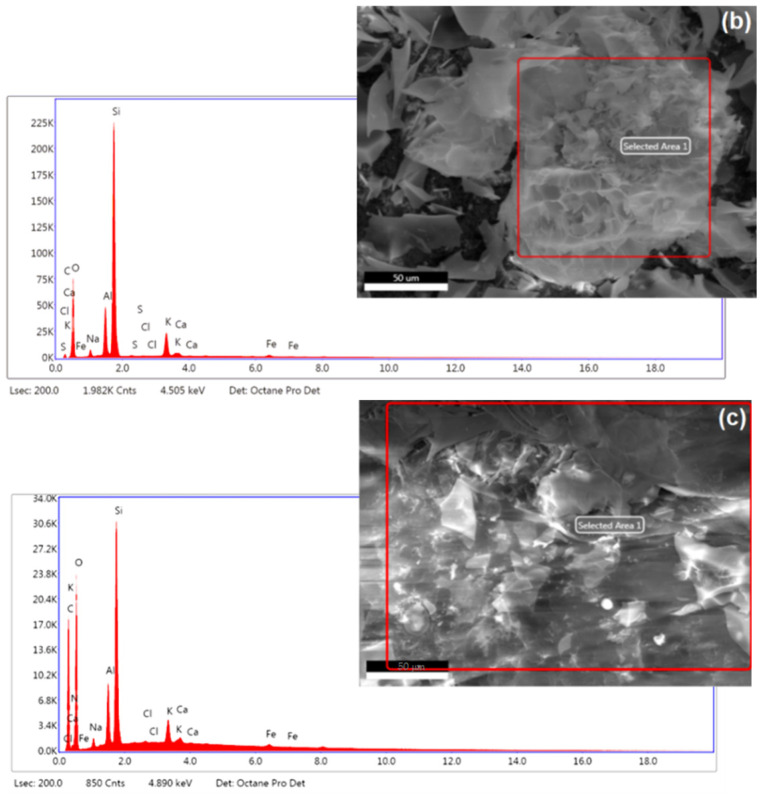
Chemical distributions of elements SEM–EDS of chitosan (**a**), perlite (**b**), and chitosan/perlite (**c**) samples.

Figure 6 shows the chemical distributions of elements in the individual material samples, with an apparent similarity in element distribution for the perlite and chitosan/perlite systems in the analysed areas. This leads to the conclusion that, despite the threefold higher proportion of chitosan in the sample, perlite largely shapes the microstructural properties of the carrier. This was previously observed by analysing the BET surface area (see Figure 4 and Table 3).

**Figure 7:**
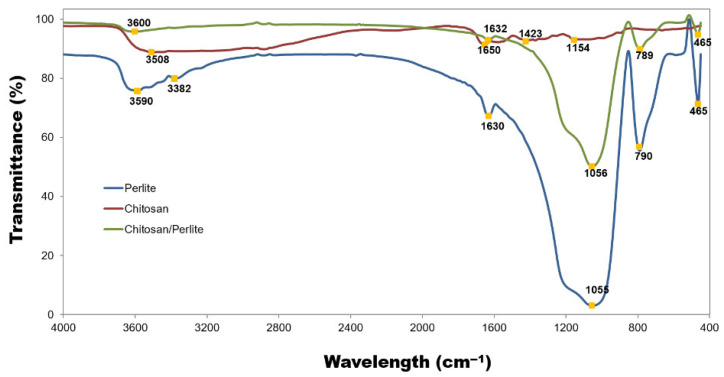
FTIR analysis of the chitosan, perlite, and chitosan/perlite samples.

The FTIR spectrum of chitosan (red line) reveals a broad absorption band centred at 3508 cm^−1^, which can be attributed to –OH stretching vibrations linked to intermolecular hydrogen bonding (see Figure 7. The stretching N–H peaks from the primary amine and amide II also overlap in the same region (3500–3300 cm^−1^). Another band absorption characteristic of chitosan appears at 1650 cm^−1^ (amide I), 1598 cm^−1^ (–NH_2_ bending), and 1423 cm^−1^ (amide III). The peak for the asymmetric stretch of C–O–C was identified at 1154 cm^−1^.

In the FTIR spectrum of perlite, characteristic absorption bands can be observed at 465 cm^−1^ and 790 cm^−1^, corresponding to the stretching vibrations of Si–O bonds. A prominent band around 1055 cm^−1^ is associated with the asymmetric stretching vibrations of Si–O–M linkages, where M represents either Al or Si. The peak located at approximately 1630 cm^−1^ is attributed to the bending vibrations of O–H groups in water molecules. Additional bands related to water are discernible at 3382 cm^−1^ and 3590 cm^−1^. Notably, the spectral profile of perlite shows a strong resemblance to that of the Ch/P composite material. These FTIR findings are broadly consistent with those reported in previous studies [19,43].

#### 3.1.4. DSC

**Figure 8:**
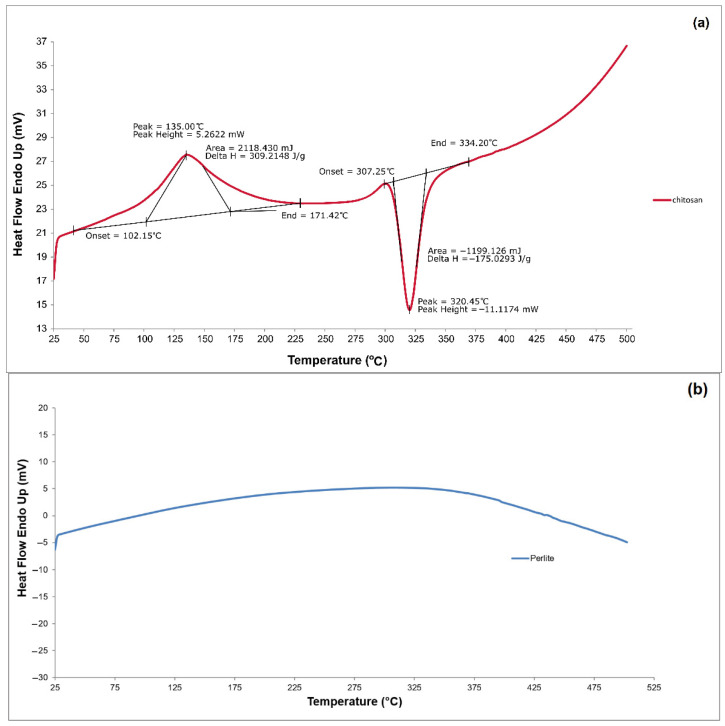

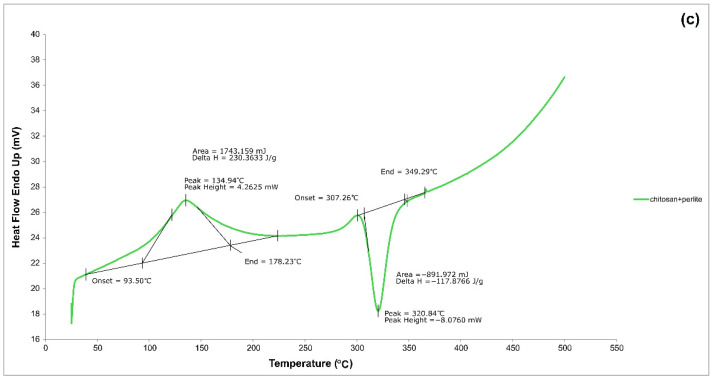
DSC thermograms of chitosan (**a**), perlite (**b**) and chitosan/perlite (**c**) samples.

Differential scanning calorimetry (DSC) is an analytical method that enables the monitoring of thermal transitions by measuring the differences in heat flow between a sample and a reference material under controlled temperature conditions. In this study, DSC was employed to evaluate the thermal stability of the microbial carrier and its individual components. The results are illustrated in Figure 8a–c.

Figure 8a shows a thermogram of a chitosan sample. It first shows a broad endothermic peak at 135 °C (also called dehydration temperature, TD) due to the evaporation of residual water bound to the hydrophilic groups of chitosan or solvent. This peak starts at about 102.15 °C and ends at about 171.42 °C. It is worth adding that, in the solid state, chitosan has a strong affinity for water and, as a result, can also undergo hydration. An endothermic peak may suggest that the bound water may not have been completely removed during drying.

A stable heat flow was observed in the chitosan sample, which showed another thermal effect (see Figure 8a): an exothermic peak at 320.45 °C attributed to the thermal degradation of chitosan (depolymerisation, saccharide ring dehydration, and decomposition of deacetylated and acetylated chitosan units). The results are consistent with those reported by other researchers [44].

The DSC curve corresponding to the perlite sample proves the high thermal resistance of this material (see Figure 8b), which confirms the results of thermal studies, including thermogravimetric differential thermal analysis (TG/DTA), carried out by other researchers [45]. The thermogram showed no peaks indicating the degradation of perlite throughout the entire temperature range used in the analysis. In contrast, the DSC thermogram of the chitosan/perlite system, shown in Figure 8c, is very similar to that of pure chitosan. The endothermic and exothermic peaks appear at similar temperature ranges and are usually responsible for thermal transformations occurring in chitosan. Therefore, the thermal properties of the tested carrier are determined by the material less resistant to elevated temperatures, which has a threefold greater weight proportion. The presence of perlite appears to have no effect and generally does not change the thermal parameters, most likely due to its insufficient proportion.

Thermal analysis of the chitosan/perlite carrier proved its thermal stability over a wide temperature range, including at thermophilic and mesophilic anaerobic digestion temperatures.

### 3.2. Bacterial Community Abundance and Composition

**Figure 9:**
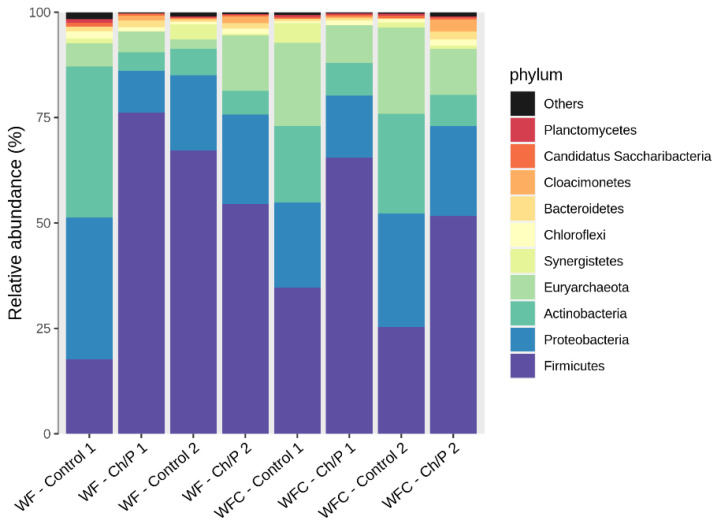
Phylum-level taxonomic composition revealed by 16S rRNA gene analysis.

The sequencing data obtained from the bacterial community analysis identified a total of 240 amplicon sequence variants (ASVs). These were classified into 157 genera, belonging to 84 families, 45 orders, 24 classes, and 11 phyla. Among the most dominant families were Clostridiaceae and Methanotrichaceae. At the phylum level, *Firmicutes* were the most prevalent, followed by *Proteobacteria* and *Actinobacteria* (see Figure 9). The comparative analysis of the samples allowed for the assessment of the content of individual taxa throughout the experiment. Similar results were obtained in studies using the diatomaceous earth/peat (DEP) cell carrier [9]. Representatives of the phyla *Proteobacteria*, *Chloroflexi*, and *Bacteroidetes* are recognised for their functional roles in wastewater treatment processes [46]. Moreover, *Firmicutes* are known for their ability to decompose a wide variety of organic compounds typically found in municipal sewage sludge [8,9].

Before the WF experiment (WF–control 1), Firmicutes (15.59%) and Proteobacteria (11.32%) were the most abundant. The addition of the carrier (WF–Ch/P 1) resulted in an increase in the bacteria from the first group to 74.13% and a decrease in Proteobacteria to 2.08%. After the experiment, the control sample (WF–control 2) remained the richest in Firmicutes (62.57%) and Proteobacteria (6.33%). The amount of bacteria from the phyla Actinobacteria, Bacteroidetes, Campylobacterota, Chloroflexi, Euryarchaeota, Planctomycetes, and Proteobacteria decreased. However, Firmicutes, Synergistetes, and Thermotogae increased during the experiment. The addition of the carrier (WF–Ch/P 2) resulted in an increased number of bacteria from taxa Actinobacteria, Bacteroidetes, Chloroflexi, Euryarchaeota, Planctomycetes, and Spirochaetes, when compared to control sample at the same stage of the experiment (WF–control 2). During the experiment, the sample with the carrier (WF–Ch/P 2) consistently maintained the highest amount of Firmicutes bacteria; however, its content decreased to 51.12% compared to the start of the experiment (WF–Ch/P 1). There was, however, an increase in the amount of bacteria of the phylum Euryarchaeota (2.72-fold increase) and Proteobacteria (2.26-fold increase). The increase in the abundance of the Euryarchaeota and Proteobacteria due to addition of Ch/P carrier to the AD process suggests significant alterations in the microbiome structure, enhanced methanogenesis, and accelerated organic matter degradation.

Euryarchaeota is a phylum of archaea that includes methanogens, which play a crucial role in the final stage of AD, namely the conversion of precursors into methane. The observed substantial increase in the abundance of these taxa implies that the carrier material supports the enhancement of methanogenesis, predominantly via direct interspecies electron transfer (DIET) pathways [47].

DIET, in more detail, is a metabolic process in which microorganisms exchange electrons directly, bypassing soluble electron carriers such as hydrogen or formate. DIET supports syntrophic relationships, particularly between different species of bacteria and archaea, by enabling efficient energy sharing and resource utilisation. Recent studies have shown that the efficiency of DIET can be enhanced by the presence of conductive or semi-conductive materials, which facilitate direct electron exchange between microbial partners [48]. Materials such as biochar, activated carbon, or certain types of modified clays have been demonstrated to promote these interactions by serving as electron conduits. Similarly, composites incorporating chitosan and perlite may influence microbial colonisation and electron transfer processes through their physicochemical and surface properties. For this process, symbiotic bacteria are crucial, as they form the metabolic partnerships necessary for electron transfer. These bacteria are divided into two main groups: (i) electron-donating bacteria—these organisms oxidise organic substrates (e.g., acetate or ethanol) and transfer the electrons to electron-accepting partners—and (ii) electron-accepting bacteria or archaea—these partners typically reduce terminal electron acceptors, such as carbon dioxide to methane, in the case of methanogens. Key genera involved in DIET include *Geobacter* and *Methanothrix* (formerly *Methanosaeta*) [49].

Considering the previously reported results in this study regarding the increased abundance of *Methanotrichaceae*, it can be assumed that the rise in Euryarchaeota is due to the intensification of acetoclastic methanogenesis via DIET, where electrons are transferred directly from syntrophic bacteria, such as *Geobacter* (an anaerobic bacterium belonging to the Proteobacteria group), to methanogenic archaea without the mediation of hydrogen. The reinforcement of the shift away from interspecies hydrogen transfer (IHT) mechanisms under the influence of the added carrier material indicates that microbial interactions in the anaerobic degradation process increasingly rely on DIET rather than the traditional IHT pathway [50].

In turn, Proteobacteria constitute a highly diverse group of bacteria, including both fermentative and electroactive species. Their 2.26-fold increase in fermentation trials with the tested additive suggests the following beneficial effects: (i) Growth of *Geobacter* (*Deltaproteobacteria*)—key bacteria involved in DIET. *Geobacter* are well-known electroactive syntrophic bacteria capable of directly transferring electrons to methanogens (*Methanothrix*). (ii) Enhanced hydrolysis and fermentation by other Proteobacteria. Certain Proteobacteria genera can contribute to the hydrolysis and fermentation of food waste components, increasing the availability of precursors for methanogenesis. Improved fermentation promotes more efficient nutrient utilisation by syntrophic bacteria and methanogens, thereby intensifying the overall AD process [47].

The combined increase in Proteobacteria (particularly Geobacter and fermentative species) and Euryarchaeota (Methanothrix) suggests that the chitosan/perlite carrier (3:1) improves biofilm formation and supports electron transfer within the DIET process, as well as accelerating the conversion of organic substrates into methane.

**Figure 10:**
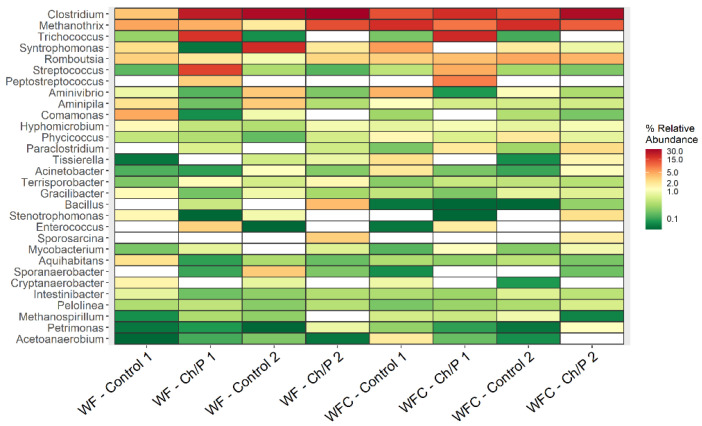
Genus taxa composition revealed by the metataxonomic analysis of the 16S rRNA gene.

*Clostridium* emerged as the dominant genus, being detected across all analysed samples, with *Methanothrix* following in relative abundance (see Figure 10). In each experimental layout, the *Clostridium* content increased when the sample was compared with the carrier against the control. The same was observed for *Mycobacterium*, *Paraclostridium*, *Pelolinea*, and *Pseudoxanthomonas*. An increase in 51 classified genera was observed in the WF–ChP 1 sample, 42 in the WF–ChP 2 sample, 42 in the WFC–ChP 1 sample, and 38 in the WFC–ChP 2 sample, in comparison to the control samples. During the experiment, 43 bacterial genera increased in the WF layout and 49 in the WFC layout. It is worth mentioning that many sequences unclassified at the genus level were also found in our research. The highest amount of such ASVs was present in the WF–ChP 2 (45.83%) and the WF–Control 2 sample (45.05%). In the majority of samples, the unclassified bacterial sequences were predominantly affiliated with the Proteobacteria phylum.

In reference to the sequencing results mentioned above, it was concluded that the increase in the abundance of bacteria from the genera *Clostridium, Mycobacterium, Paraclostridium, Pelolinea*, and *Pseudoxanthomonas* after the addition of the Ch/P carrier, compared to the control, may indicate significant changes in the anaerobic fermentation microbiome. *Clostridium* and *Paraclostridium* are anaerobic bacteria capable of fermenting sugars, proteins, and fats, which are key in the early stages of food waste degradation. Their increased abundance may suggest improved conditions for hydrolysis. Their metabolic products (e.g., organic acids, hydrogen) could fuel subsequent processes, including methanogenesis [51]. Although *Mycobacterium* is primarily associated with pathogens, some environmental species participate in the biodegradation of fats, lignin, and aromatic compounds. On the other hand, *Pelolinea* are bacteria belonging to *Chloroflexi*, commonly found in sewage sludge and associated with syntrophic processes. Their growth may indicate that the addition of the carrier has positively affected the development of syntrophic communities, as well as DIET. *Pseudoxanthomonas* are facultative anaerobic bacteria that can be involved in the breakdown of fatty acids and other fermentation products. Their growth suggests better conversion of organic substrates, which may positively influence the microbiological balance and the efficiency of anaerobic fermentation [51]. To summarise, the noted rise in the abundance of these genera following the addition of the investigated carrier suggests an enhancement of hydrolysis, fermentation, and syntrophic interactions, which is expected to contribute to greater efficiency of the anaerobic digestion process.

The MetaStat analysis highlights statistically significant differences between the control groups and the samples supplemented with the carrier. The variations in genus composition among all analysed samples are depicted in Figures S1–S6. Each diagram presents up to 30 taxa. A comparative analysis between samples WF–Control 1 and WF–Ch/P 1 showed differences in the abundance of 119 taxa (higher content of 47 bacteria in the sample with carrier) (Figure S1). This analysis confirms that the carrier stimulates cell proliferation and alters the microbiome composition. In comparison, fewer statistically significant differences (81) were observed between WF–Control 2 and WF–Ch/P 2 (Figure S2). Both samples come from the final stage of biodegradation. In both samples, limited bacteria access to the carrier was noted.

In the WFC–Ch/P 1 sample, a higher amount of 37 bacteria genus was observed compared to the WFC–Control 1 sample. The highest differences were observed for *Trichococcus*, *Macellibacteroides*, *Lacticaseibacillus*, *Longilinea*, *Limosilactobacillus*, *Falcatimonas*, *Pseudobutyrivibrio*, *Gemmobacter*, *Brevilactibacter*, *Uruburuella*, *Eubacterium*, *Erysipelatoclostridium*, *Veillonella*, *Enterococcus*, *Peptostreptococcus*, *Mycobacterium*, *Clostridium*, *Anaeroarcus*, *Desulfobulbus*, *Paraclostridium*, *Ligilactobacillus*, and *Streptococcus* (Figure S3).

Among the 30 bacterial genera exhibiting increased abundance in the sample with the added carrier (WFC–Ch/P 2) compared to the control sample (WFC–Control 2), the following genera showed the highest statistical significance: *Dietzia*, *Gemmobacter*, *Propionicimonas*, *Aminobacterium*, *Bacillus*, *Alcaligenes*, *Clostridium*, *Mycobacterium*, *Paraclostridium*, *Acinetobacter*, *Paenalcaligenes*, *Stenotrophomonas*, *Thermomonas*, *Tissierella*, *Sporosarcina*, *Fermentimonas*, *Massilibacterium*, *Pseudomonas*, *Petrimonas*, and *Sporanaerobacter* (Figure S4).

Over the course of the experiment, there were changes that led to the differences in the content of 72 taxa in the WF samples. More taxa (39), whose abundance was statistically significantly higher, were observed in the sample taken near the final stage of the experiment (WF–Ch/P 2) (Figure S5). Almost the same observations were made for the WFC experiment (Figure S6).

The alpha and beta diversity metrics calculated based on 16S rRNA sequencing data are shown in Table 5 and Table 6.

The analysis identified a total of 342 unique bacterial ASVs, the majority of which were detected in the control samples. The core microbiome was composed of 291 ASVs (Figure S7a–f). There were 47 unique ASVs in the WF–Ch/P 1 sample (Figure S7a), 32 in the WF–Ch/P 2 sample (Figure S7b), 33 in the WFC–Ch/P 1 sample (Figure S7c), and 26 in the WFC–Ch/P 2 sample (Figure S7d), compared to the control samples. The addition of different carriers resulted in 90 ASVs in the core microbiome at the start of the experiment (Figure S7e) and 82 ASVs at its end (Figure S7f).

### 3.3. Physico-Chemical Analysis of Sludge Samples and Biogas Performance

The utilisation of organic substrates with diverse compositions in waste-fed biogas plants presents the potential for achieving high process efficiency; however, it also necessitates continuous monitoring of the fermentation process. Such oversight enables effective control and optimisation of anaerobic digestion. During the decomposition of biomass, especially materials rich in fatty carbohydrates, VFAs can be rapidly released in the first stage, which often results in an acidified environment [27].

In the case of the presented experiment, the process was stable, as indicated by the recorded pH values, VFA concentration, and VFA/TA ratio. The recorded pH ranged from 6.9 to 7.6, with a slight increase in pH for mixtures with added cheese waste as a result of casein decomposition [29]. VFA concentration was obtained at relatively low values ranging from 1050 mg L^−1^ to 1970 mg L^−1^, with higher values corresponding to samples with the cosubstrate system and the addition of the carrier. On the other hand, the VFA/TA ratios were very similar, both for samples with and without the carrier, ranging from 0.26 to 0.43, confirming process stability.

**Figure 11:**
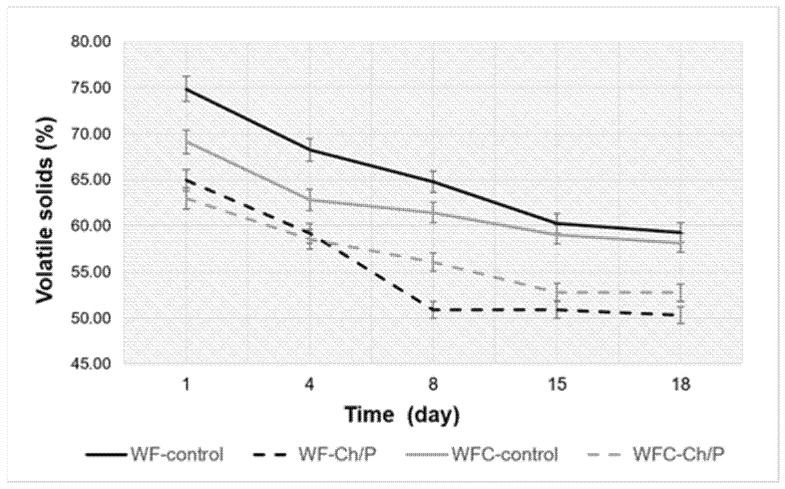
Temporal variations in volatile solids content during the anaerobic digestion process.

Quantitative changes in organic matter over time were also measured for in-process samples (from the four bioreactors studied) (see Figure 11). It was confirmed that during the fermentation of the mixtures, there was a loss of organic matter (with the release of biogas), which translated into a decrease in the content of volatile solids in the total solids of the fermented material.

Due to the additional materials in the mixtures, a higher organic matter content was observed in the control samples than in those with carrier addition. In the WF variant, a decrease in VS content of approx. 15% was observed, similar to the control and test samples. In contrast, in the WFC variant, the same difference was approx. 11%, also for both samples. The amount of decomposed organic matter thus depended on the type of substrate rather than the carrier’s presence.

**Figure 12:**
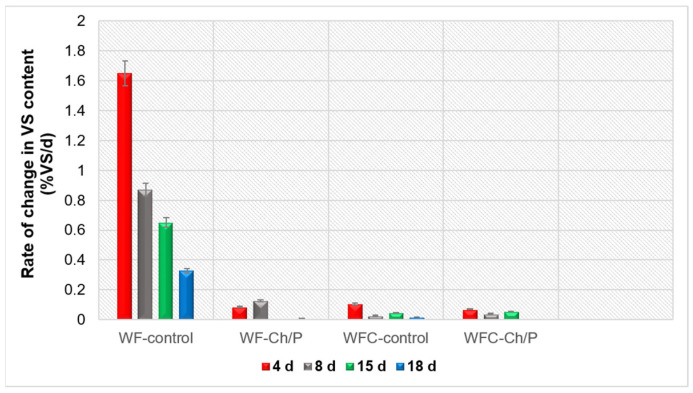
Rate of changes in the content of volatile solids in samples collected during anaerobic digestion.

Using samples from the bioreactors tested, the rate of changes in the organic matter content of the mixtures tested was also analysed (see Figure 12).

It was found that this parameter was higher for the control samples than for the test samples, which could be related to the addition of the carrier to the treatment variants. At the same time, a higher rate of change was found for the WF variant than for the WFC variant, which could be related to the type of substrate used for fermentation and is consistent with the greater decrease in VS content in the mixture shown earlier.

**Figure 13:**
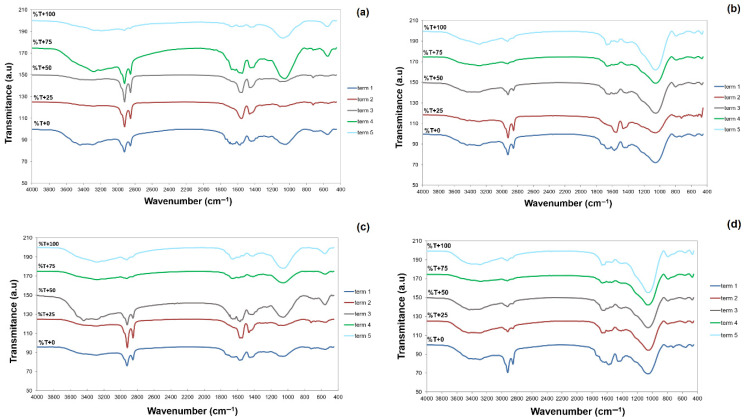
FTIR spectra of samples collected during anaerobic digestion from batches: (**a**) WF–control, (**b**) WF–Ch/P, (**c**) WFC–control, (**d**) WFC–Ch/P.

FTIR spectroscopy was employed to monitor qualitative changes in the chemical composition of the bioreactors throughout the process, as shown in Figure 13a–d.

When interpreting the accompanying FTIR spectra, it should be emphasised that the basic material of the batches is stabilised sewage sludge (acting as an inoculum). Hence, the shape of the curves shows great similarity to the spectra of pure sewage sludge. It should also be stressed that with such a small (yet standard for the organic load of an anaerobic bioreactor) proportion of other organic materials (substrates), the material could not undergo a reaction. Hence, the spectral shape for all four samples is essentially identical. Differences concern the size of the characteristic bands, which may indicate different kinetics of decomposition of the individual substances in the bioreactors. The biodegradation rate may be influenced either by the proportion of the carrier or, as concluded before (Figure 12), by the arrangement of the substrates undergoing the process. Matheri et al. (2020) confirmed that the chemical composition of sewage sludge samples influences the shape of FTIR spectra, which simultaneously impacts the degradation process [52].

Sewage sludge is primarily composed of proteins, carbohydrates, and lipids; however, following anaerobic digestion, the content of carbon-based compounds may be significantly reduced. The FTIR spectra of sewage sludge (Figure 13a–d) revealed a broad and intense absorption band in the 3200–3600 cm^−1^ region, corresponding to O–H and N–H stretching vibrations. Such vibrational bands indicate the presence of alcohols, carboxylic acids, and amides/amines, predominantly originating from the organic constituents of sewage sludge. A distinct peak at approximately 3400 cm^−1^ observed in the dried samples suggests the presence of alcohols, phenols, ethers, and acids. This spectral region showed comparable intensities across the samples and exhibited a degree of flattening over time. Additionally, two bands around 2900 cm^−1^ and 2800 cm^−1^ were particularly pronounced at the initial sampling points, appearing as sharp peaks. These bands are attributed to asymmetric and symmetric C–H stretching vibrations, respectively. The subsequent reduction in their intensity—most evident in Figure 13b,d (samples containing the carrier)—reflects the decomposition of aliphatic chains in carbohydrates and lipids. In contrast, this change was least noticeable in the control sample with wafer waste only. These observations suggest a beneficial synergistic effect resulting from the combined application of the carrier and cosubstrate in the system [18].

In addition to fats and carbohydrates, derived from both the inoculum (sludge) and the substrates, proteins were also present, as evidenced by the amide I and amide II bands. These bands, which gradually diminished in the spectra over time, were recorded at 1650 cm^−1^ and 1546 cm^−1^ and correspond to C=O stretching and N–H bending vibrations, respectively. The presence of cellulose—characteristic of sewage sludge—was confirmed by C–O stretching vibrations between 1000 and 1200 cm^−1^, consistent with the findings of Yang et al. (2007) [53].

Furthermore, minor peaks observed between 600 and 900 cm^−1^ indicate the presence of aromatic compounds. The overall spectral characteristics are in agreement with previously reported data [54].

The shape of the spectra, whose changes during the process particularly concern carbon-related functional groups, indicates the dependence of the degradation rate on the presence of the carrier and the choice of cosubstrate; in the case analysed, the combination of confectionery and cheese waste as a balanced carrier for the cells is particularly important [27].

The biogas yields show a positive effect of both the addition of the carrier and the combination of confectionery and dairy waste in the cosubstrate system.

**Figure 14:**
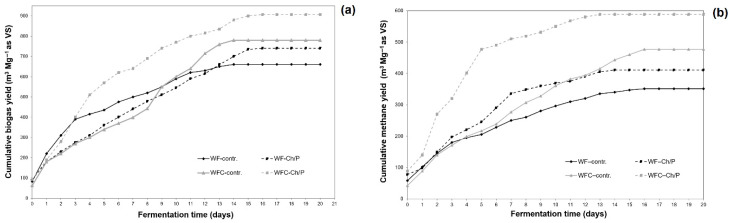
Cumulative (**a**) biogas and (**b**) methane production curves normalised to the volatile solids (VS) content of the tested samples.

Biogas and methane production curves confirming the stable course of the fermentation process are presented below (see Figure 14a,b).

For the fermented sample WF–control and WF–Ch/P, the addition of a cell carrier based on Ch/P (3:1) contributed to a 12.05% increase in biogas generation efficiency (Table 7). This addition, as an effective support for methanogenic cells, also increased the biogas methane content (from 53.2% to 55.5%), indicating an increase in the decomposition rate of organic matter. Combining the cosubstrate used in the system with porous and compatible microbial carriers improves process efficiency, as proved in another study by the same authors [27]. In the WFC–control and WFC–Ch/P systems, there was a 16.20% increase in biogas yield. Compared with other carriers previously tested by the same researchers, the values obtained are comparable or slightly lower [8,31]. Nevertheless, both currently discussed and previously presented carrier materials are worthy of consideration due to their properties, favourable impact on process efficiency, accessibility, and price.

The methane volume produced from the WF–control variant, expressed per unit of volatile solids (VS), amounted to 351.19 ± 14.03 m^3^·Mg^−1^ (refer to Table 7), which rose to 410.74 ± 16.40 m^3^·Mg^−1^ upon the introduction of the carrier. The amount of methane obtained from the control cosubstrate sample was higher, reaching 476.84 ± 19.02 m^3^·Mg^−1^ (VS). The addition of the carrier to the WFC–Ch/P system further enhanced methane productivity, achieving 588.55 ± 23.49 m^3^·Mg^−1^ (VS). In the case of the WFC cosubstrate system, a synergistic effect was observed, confirming the authors’ earlier findings [27]. Correspondingly, the methane content in biogas ranged from 53.2% (±2.2%) for WF–control to 64.9% (±2.7%) for WFC–Ch/P, further supporting the superior performance of the co-fermented variant.

The expanded uncertainties (*U*) presented in Table 7 range from approximately ±26.15 to ±35.88 m^3^·Mg^−1^ VS for biogas yield and from ±14.03 to ±23.49 m^3^·Mg^−1^ VS for methane yield. These values correspond to relative uncertainties of approximately 4–6%, which are acceptable for laboratory-scale experiments involving biological processes, where certain variability is inherent. The presented results clearly indicate that the uncertainty intervals do not overlap to an extent that would hinder the interpretation of differences between variants. This provides strong evidence that the applied methodology offers a sufficient level of precision for comparative analysis, and that the observed differences in performance are statistically meaningful, despite the associated uncertainties.

Moreover, the quantitative assessment of uncertainty—expressed as expanded uncertainty at a 95% confidence level—further confirms the reliability of the obtained results. The magnitude of the observed differences consistently exceeds the calculated uncertainties, thereby supporting the validity of conclusions regarding the effects of carrier addition and substrate formulation on biogas production efficiency.

The results of the BMP tests for the Ch/P carrier and its individual components were not presented, for both substantive and practical reasons. The justification lies in the fact that perlite does not undergo biodegradation, while chitosan, under the specific conditions of the AD process maintained in this experiment, also remains largely non-degradable. The volume of biogas obtained in the trial test involving chitosan, under comparable conditions in three replicates, ranged from 1.23 m^3^ to 1.41 m^3^. These values are negligible in comparison to the amount of biogas produced from the analysed substrates (Table 7). It should be emphasised that the high-molecular-weight chitosan used in this study (as detailed in Section 2.1), characterised by a low degree of deacetylation (DD ≤ 70%), exhibits greater resistance to microbial hydrolysis and, consequently, reduced susceptibility to biodegradation [55]. Its stability is favoured by mesophilic conditions, a stable pH of approximately 7 (as it dissolves in acidic environments), and, most importantly, a short retention time (below 60 days), which was upheld in this study. The resistance of chitosan to enzymatic degradation by microorganisms, particularly under anaerobic conditions, is further supported by the presence of amino groups and its positive charge [56].

### 3.4. Literature Review and Discussion

In the context of anaerobic digestion, as investigated in the present study, the observed increase in methane production resulting from the addition of chitosan may be attributed not only to its highly developed surface area, but also to its distinct chemical structure and the functional properties of this compound [10]. Yin and Chen (2022) demonstrated a significant influence of chitosan (CTS) on the anaerobic digestion of waste-activated sludge (WAS) [16]. Their findings revealed a positive correlation between the CTS content and the methane production potential of WAS. Notably, the introduction of 30 g/kg of CTS into the total suspension increased the cumulative methane yield from 215 ± 1.52 to 272 ± 1.83 mL g^−1^ of volatile suspended solids. It should be emphasised that the research conducted by the present authors was carried out under different process conditions than those described in the article, specifically in a continuous bioreactor operation mode over a 100-day period [16]. Under these conditions, chitosan subjected to anaerobic digestion underwent significant biodegradation. Nevertheless, the observed phenomena and resulting conclusions are of scientific significance. According to the cited researchers [16], the positively charged amino groups in chitosan neutralise the hydroxyl and carboxyl groups of extracellular polymeric substances, which reduces the negative charge of the sludge surface and promotes sludge agglomeration. Chitosan also slows down hydrolysis and reduces acidification by immobilising hydrolase and acidulase enzymes, which stabilises anaerobic digestion. On the other hand, this additive flocculates humus to avoid its interference with electron transfer, thereby increasing the activity of coenzyme F420 and methanogenesis.

Chitosan effectively functions as an adsorbent, biocatalyst, or carrier, often in the form of composites with other materials. The combination of chitosan with other natural materials imparts specific functional properties, enhancing mechanical strength and electronic conductivity, increasing resistance to degradation, and providing additional surface area for microbial adhesion. Numerous literature reports confirm the significance of research in this field [57,58]. Tetteh et al. (2022) used chitosan in chitosan–magnetite (nCM; magnetite Fe_3_O_4_) and chitosan–titania–magnetite (nCTM) systems as biocatalysts for bioremediation of wastewater with methanogenic activity enhancement [18]. The authors concluded that nCTM is a very potent biocatalyst stimulating biogas production and methanogenic activity with a 75% degradation rate. Their study, on the other hand, showed that the development of nanobiocatalysts could provide a viable alternative to conventional anaerobic biogas production in wastewater treatment. In contrast, Nie et al. (2023) explained the mechanism of action of a chitosan–magnetite system, chitosan–Fe_3_O_4_ [17]. Sediment analysis revealed that chitosan–Fe_3_O_4_ enhanced the secretion of proteins and humic substances within the extracellular polymeric substances (EPS), thereby increasing the system’s electron transfer activity by 71.4%. Microbial community profiling further indicated that chitosan–Fe_3_O_4_ contributed to the proliferation of *Peptoclostridium*, while *Methanosaeta* played a role in facilitating the DIET (direct interspecies electron transfer) mechanism. Consequently, the addition of chitosan–Fe_3_O_4_ [17], as well as chitosan alone [16] or magnetite [18], can support the establishment of DIET pathways, thereby promoting stable methanogenic activity. These materials enhance the abundance of methanogenic archaea, which naturally contributes to elevated methane yields [9]. Key aspects related to DIET, including those relevant to the present study, are further discussed in Section 3.2. It is therefore evident that the action of chitosan is multifactorial, involving a range of molecular mechanisms. From the perspective of enhancing the efficiency of biogas production, further research into anaerobic digestion systems incorporating chitosan appears to be both justified and necessary.

In turn, perlite has been widely reported as a microbial carrier with various functional applications, particularly in the construction industry. Microbial mineralisation enables self-diagnosis. The direct immobilisation of active microorganisms within the cement matrix facilitates interaction between the microbial cells and the material, positively influencing both bacterial viability and the durability of the matrix. However, the alkaline environment and the dense internal structure necessitate additional bacterial protection, which is covered by a number of scientific studies. For example, Jiang et al. (2020) investigated the effect of expanded perlite (EP) wrapped in various materials as a carrier for bacteria and nutrients to correct cracks and improve concrete regeneration [59]. EP particles were found to improve the healing capacity of concrete cracks after wrapping. This effect was particularly noticeable when EP particles immobilised by bacterial spores were wrapped with a low-alkaline, potassium–magnesium phosphate-based material. This result was also confirmed in water permeability tests. On the other hand, Yan et al. (2024) confirmed, among other things, that the number of microbial colonies declines with decreasing freezing temperatures and that the smaller particle size of a perlite carrier exacerbates the adsorption effect and, at the same time, the protective effect on microorganisms in alkaline environments [60]. When it comes to perlite as a microbial carrier in biogas production, it should be noted that it has rarely been used in this application so far. However, as mentioned earlier, the feasibility of this application has been proven. Moreover, it has significant effects, including increased immobilisation of microorganisms and accelerated substrate degradation, both under aerobic and anaerobic conditions [23].

If the chitosan/perlite systems are taken into account, these materials have thus far been primarily developed for applications in adsorption [24,25,59] and catalysis [26], as outlined earlier in this article. Vijaya et al. (2010) designed a novel biosorbent by coating chitosan onto perlite (CCP) [24]. FTIR analysis was employed to identify the functional groups responsible for fluoride adsorption. The adsorption properties of CCP towards fluoride were evaluated under both batch equilibrium and column flow conditions. Sahbaza and Acikgoz (2017) [61] investigated the removal of the dye Ostazin Black NH (OB) from aqueous solution using chitosan-coated perlite beads through batch adsorption, taking into account various parameters affecting removal efficiency. The findings confirmed that chitosan-coated perlite beads represent a viable, cost-effective, and environmentally sustainable adsorbent for dye removal. Noteworthy research was also carried out by Demirçivi (2018) [25], who synthesised and characterised a novel composite adsorbent—zirconium(IV)-doped, immobilised, cross-linked chitosan/perlite (Zr(IV)-CS-PT)—in order to enhance the adsorption performance of chitosan for Acid Orange II (AOII). Desorption and reuse experiments demonstrated that Zr(IV)-CS-PT is a reusable, cost-efficient material with high adsorption capacity.

The decision to combine chitosan and perlite in research is underpinned by several scientific hypotheses rooted in their complementary chemical, physical, and biological properties. These hypotheses suggest that the hybrid system could exhibit synergistic effects that surpass the capabilities of the individual components. Key scientific rationales include the following: (i) hypothesis of synergistic material interaction—by combining these materials, it is hypothesised that the hybrid system would achieve an enhanced adsorption capacity due to both chemical interaction and physical reinforcement; (ii) hypothesis of enhanced mechanical and thermal stability—the incorporation of perlite would improve the mechanical robustness and thermal stability of chitosan-based materials, broadening their range of practical applications; (iii) hypothesis of complementary functionalities—the combination could create a material that merges the bioactivity of chitosan with the structural advantages of perlite; (iv) hypothesis of improved adsorption and catalytic properties—the hybrid material would provide an optimal environment for adsorption or catalytic processes, where perlite ensures efficient transport and chitosan provides chemical affinity; (v) hypothesis of hybrid material tailoring—the creation of a material with specific characteristics, such as selective adsorption of pollutants, immobilisation of enzymes, or controlled release of bioactive compounds; (vi) hypothesis of sustainability and waste utilisation—combination could create a sustainable material, aligning with principles of green chemistry and circular economy. Both chitosan and perlite are derived from natural or readily available sources, with chitosan often obtained from crustacean shell waste and perlite mined in abundance. Their combination creates a sustainable material, aligning with principles of green chemistry and circular economy.

In summary, the development and use of microbial carriers in AD offer significant prospects for enhancing the efficiency and stability of biogas production. The increased surface area of porous carriers facilitates the formation of biofilms in which microorganisms can aggregate and interact, enhancing their metabolic activity. This results in faster and more efficient decomposition of organic matter. In addition to the zeolites mentioned in the introduction, the following have also been tested as potential anaerobic digestion microbial carriers: granulated polymeric support [poly(acrylonitrile-acrylamide)], rubberised-coir, ‘magnetite+foam carrier’ system, magnetite-modified zeolite, pumice, nanofiber membrane, and biochar. Liu et al. (2017) focused exclusively on biofilm carriers, testing polypropylene, polyester, polyamide, and polyurethane fibres, with the most favourable results obtained using polypropylene fibre [5]. Conversely, Zamrisham et al. (2024) recently studied the integration of lava rock, red clay, and ceramic bio rings as support carriers in the anaerobic treatment of landfill leachate with liquefied food waste [62]. Research on carriers used in the anaerobic biodegradation of organic waste from various sources is therefore widespread, highlighting the importance and considerable potential of such materials. To date, the authors of this study have tested carrier systems composed of silica/lignin, diatomaceous earth/peat, and granular polylactide; Pilarska et al. [8,9,32] successfully selected complementary materials in terms of properties and functions performed in the process.

Furthermore, it should be noted that recent advances in materials science have led to a trend of integrating materials into functional systems that incorporate natural materials, synthetic polymers, and nanomaterials. Each type of carrier offers unique properties suitable for specific AD applications. Research is also currently underway to develop dedicated microbial consortia that can be immobilised on the carriers. These consortia are optimised to degrade specific types of feedstocks, increasing the overall AD efficiency and effectiveness. It is also worth mentioning that the combination of microbial carriers with advanced monitoring and control systems offers a considerable opportunity to optimise anaerobic digestion in real time. Sensors and automation can provide continuous feedback on microbial activity, enabling dynamic adjustments to operating conditions.

The chitosan/perlite (3:1) composite, as investigated in this study, has demonstrated particularly advantageous properties for potential industrial application. Its high porosity, biocompatibility, and surface functionality support robust microbial colonisation and facilitate stable syntrophic interactions, including those involved in DIET. In addition, the chitosan component offers mild antimicrobial properties, which may selectively reduce undesirable microbial populations, while perlite enhances mechanical stability and ensures uniform distribution within the reactor. The material’s physicochemical profile also makes it compatible with process intensification strategies, such as high-loading systems and hybrid digester designs. Pilot-scale studies could help determine the optimal granulation, packing density, and dosing strategies to maximise its performance under varying operating conditions.

However, it is important not to overlook the risk factors in transferring a process from the laboratory to the industrial scale. In addition to the known factors that can impair the efficiency of a technical-scale process—such as variable substrate composition and impurities, increased risk of substrate unreactivity and release of process inhibitors (sulphur compounds, ammonia), different batch compositions compared to model conditions, hydraulic retention time and, ultimately, plant failure—other problems may also arise. These include primarily economic considerations—the materials supporting the process must be cheap and available and applied in optimal amounts that do not inhibit it. Additional scale-up challenges include ensuring uniform dispersion of the carrier material throughout large-scale digesters, preventing clogging or sedimentation, and verifying its long-term mechanical and chemical stability under industrial conditions. Consideration must also be given to the regeneration or replacement of the carrier, and to the feasibility of its integration into existing infrastructure without significant retrofitting. Furthermore, continuous monitoring of material wear and potential degradation under varying operational conditions will be crucial to assess long-term viability. Scaling up the process may also require addressing potential challenges in ensuring homogeneous microbial distribution across larger volumes, which could affect process efficiency. Lastly, strategies to minimise the environmental impact of materials over time, such as their reusability or recyclability, must be factored into the design of industrial-scale systems.

The prospects for developing and using microbiological carriers (including systems such as the studied combination of chitosan and perlite) in anaerobic digestion are promising. By enhancing microbial activity, improving process stability, and increasing methane yields, these materials can significantly increase the efficiency and sustainability of biogas production. Ongoing research, technological advances, and successful pilot projects will be key to overcoming current challenges and realising the full potential of microbial carriers in anaerobic digestion.

## 4. Conclusions

The findings of this study confirmed the functional potential of the Ch/P support material in enhancing anaerobic digestion performance. Its physico-chemical properties—including a well-developed surface area, appropriate porosity, and thermal resilience—make it a promising and environmentally sustainable material for applications in anaerobic bioprocesses. As shown by other analyses as part of the experiment, this carrier induces targeted changes in the anaerobic biodegradation environment, i.a. increasing the participation of phylum Euryarchaeota (2.72-fold increase) and Proteobacteria (2.26-fold increase). The increase in the abundance of the Euryarchaeota and Proteobacteria suggests significant alterations in the microbiome structure and enhanced methanogenesis, as well as an acceleration of the conversion of organic substrates into methane. Analysis of the removal kinetics of organic matter and FTIR indicated a dependence of the degradation rate on both the combination of substrates used and the presence of a carrier. The monitoring parameters confirmed a stable degradation. These factors resulted in an increase in biogas/methane productivity. The methane volume for the WF–control rose from 351.72 to 410.74 m^3^·Mg^−1^ (VS), whereas in the cosubstrate sample, it increased from 476.84 to 588.55 m^3^·Mg^−1^ (VS).

The essence of applying additives in the anaerobic digestion (AD) process is to enhance its stability and efficiency. This can be achieved by influencing various factors that accompany the progression of the process. The materials used can exhibit multifunctionality, and it is worthwhile to investigate them from multiple perspectives. In the case of the applied chitosan/perlite (3:1) carrier, it would be valuable to explore its dual functionality—as an effective cell carrier and as a biosorbent for potential process inhibitors. Comprehensive research facilitates the optimisation of additive design and their selection for specific applications in the AD process.

## Figures and Tables

**Table 1:** Physico-chemical properties of feedstocks and inoculum.

Mat.	pH	MU	Cond.	MU	TS	MU	VS	MU	C/N Ratio	MU	C	MU	N	MU	N–NH_4_^+^	MU	P_total_	MU
	–	(±)	(mS cm^−1^)	(±)	(wt %)	(±)	(wt %_TS_)	(±)	–	(±)	(wt %_TS_)	(±)	(wt %_TS_)	(±)	(wt %_TS_)	(±)	(wt %_TS_)	(±)
Wafers	7.02	0.39	1.62	0.08	73.24	4.49	96.81	6.12	37.50	2.07	42.37	2.27	1.13	0.06	0.36	0.02	0.15	0.01
Cheese	4.32	0.24	72.96	3.74	34.25	2.10	92.56	5.85	3.60	0.20	45.87	2.46	12.73	0.69	0.53	0.03	1.62	0.10
Inoc.	7.93	0.45	26.50	1.36	3.47	0.21	71.43	4.52	11.21	0.62	33.06	1.77	2.95	0.16	2.48	0.14	2.57	0.15

Mat.—materials, Inoc.—inoculum, MU—measurement uncertainty, Cond.—conductivity, TS—total solids, VS—volatile solids.

**Table 2:** Composition and selected physico-chemical characteristics of substrates and inoculum.

Samples	WF	MU	CE	MU	Ch/P	MU	Inoc.	MU	pH	MU	Cond.	MU	TS	MU	VS	MU
	(g)	(±)	(g)	(±)	(g)	(±)	(g)	(±)	–	(±)	(mS cm^−1^)	(±)	(%)	(±)	(%)	(±)
WF–control	9.5	0.10	–	–	–	–	835.5	8.36	7.16	0.40	62.95	3.23	4.08	0.25	73.46	4.6
WF–Ch/P	9.5	0.10	–	–	20.0	0.20	835.5	8.36	7.03	0.40	69.25	3.55	3.97	0.24	72.62	4.6
WFC–control	6.5	0.07	3.0	0.03	–	–	833.0	8.33	6.91	0.39	77.54	3.96	4.15	0.25	69.38	4.4
WFC–Ch/P	6.5	0.07	3.0	0.03	20.0	0.20	833.0	8.33	6.83	0.38	78.86	4.04	4.23	0.26	68.55	4.3

WF—wafers, CE—cheese, Ch/P—chitosan/perlite, Inoc.—inoculum, Cond.—conductivity, TS—total solids, VS—volatile solids, MU—measurement uncertainty.

**Table 3:** Textural properties.

Materials	A_BET_ (m^2^/g)	V_p_ (cm^3^/g)	S_p_ (nm)
Chitosan	0.5061	0.001441	27.366
Perlite	1.3730	0.002678	11.842
Chitosan/perlite	1.0746	0.002432	13.714

A_BET_—BET surface area; V_p_—pore volume; S_p_—pore diameter.

**Table 4:** Chemical composition of the chitosan, perlite, and chitosan/perlite samples.

Elemental Content, Weight (%)											
Material	C K	N K	O K	Na K	Al K	Si K	S K	Cl K	K K	Ca K	Fe K
Chitosan	41.10	5.22	53.55	–	0.13	–	–	–	–	–	–
Perlite	2.14	–	48.09	2.00	7.38	34.42	0.13	0.06	4.64	0.64	0.49
Chitosan/Perlite	24.43	3.56	60.66	0.79	2.27	7.20	–	0.05	0.72	0.20	0.13

**Table 5:** Alpha diversity based on the 16S rRNA sequencing data.

	Chao1	Shannon	Simpson
WF–Control 1	140	3.773	0.956
WF–Ch/P 1	117	2.994	0.88
WF–Control 2	111	2.923	0.855
WF–Ch/P 2	96	2.932	0.849
WFC–Control 1	120	3.407	0.929
WFC–Ch/P 1	111	3.154	0.903
WFC–Control 2	124	3.36	0.926
WFC–Ch/P 2	105	3.24	0.882

**Table 6:** Beta diversity assessed by Bray–Curtis analysis of 16S rRNA sequencing data.

	Bray–Curtis Index
WF–Control 1 vs. WF–Ch/P 1	0.749
WF–Control 2 vs. WF–Ch/P 2	0.466
WFC–Control 1 vs. WFC–Ch/P 1	0.532
WFC–Control 2 vs. WFC–Ch/P 2	0.468
WF–Ch/P 1 vs. WFC–Ch/P 1	0.264
WF–Ch/P 2 vs. WFC–Ch/P 2	0.228
WF–Ch/P 1 vs. WF–Ch/P 2	0.509
WFC–Ch/P 1 vs. WFC–Ch/P 2	0.451

**Table 7:** Overall biogas and methane yields.

Samples	Biogas Efficiency				Methane Efficiency				CH_4_ Content	
	(m^3^ Mg^−1^ as TS)	MU (±)	(m^3^ Mg^−1^ as VS)	MU (±)	(m^3^ Mg^−1^ as TS)	MU (±)	(m^3^ Mg^−1^ as VS)	MU (±)	(%)	MU (±)
WF–contr.	468.19	16.68	660.13	26.15	249.08	8.80	351.19	14.03	53.2	2.2
WF–Ch/P	524.61	18.69	740.08	29.30	291.16	10.28	410.74	16.40	55.5	2.3
WFC–contr.	545.21	19.42	780.42	30.86	333.12	11.76	476.84	19.02	61.1	2.5
WFC–Ch/P	633.53	22.57	906.85	35.88	411.16	14.50	588.55	23.49	64.9	2.7

MU—measurement uncertainty; contr.—control.

## Data Availability

The original contributions presented in the study are included in the article/Supplementary Materials, further inquiries can be directed to the corresponding author.

## Supplementary Materials

The following supporting information can be downloaded at: https://www.mdpi.com/article/10.3390/ma18153504/s1. Figure S1: Comparison (MetaStat analysis) of bacterial genus composition between WF–Control 1 and WF–Ch/P 1, Figure S2: Comparison (MataStat analysis) of bacterial genus composition between WF–Control 2 and WF–Ch/P 2, Figure S3: Comparison (MetaStat analysis) of bacterial genus composition between WFC–Control 1 and WFC–Ch/P 1, Figure S4: Comparison (MetaStat analysis) of bacterial genus composition between WFC– Control 2 and WFC–Ch/P 2, Figure S5: Comparison (MetaStat analysis) of bacterial genus composition between WF–Ch/P 1 and WF–Ch/P 2, Figure S6: Comparison (MetaStat analysis) of bacterial genus composition between WFC–Ch/P 1 and WFC–Ch/P 2, Figure S7: Venn diagrams prepared based on the sequencing of the 16S rRNA gene. The data shown in the diagram refer to site–specific unique ASVs and the core microbiome (a–f).

## Abbreviations

This manuscript uses the following abbreviations:

A_BET_	Brunauer–Emmett–Teller (BET) surface area
AD	Anaerobic digestion
ASVs	Amplicon sequence variants
BJH	Barrett–Joyner–Halenda method for calculating pore size distributions
CE	Cheese
DSC	Differential scanning calorimetry
EDS	Energy dispersive X-ray spectroscopy
FTIR	Fourier–transform infrared spectroscopy
MU	Measurement uncertainty
NGS	Next-generation sequencing
PCR	Polymerase chain reaction
PdI	Polydispersity index
RDP	Ribosomal Database Project
SEM	Scanning electron microscope
S_p_	Pore diameter
SSA	Specific surface area
TA	Total alkalinity
TCD	Thermal conductivity detector
TD	Dehydration temperature
TG/DTA	Thermogravimetric/differential thermal analysis
TS	Total solids
VFA	Volatile fatty acids
V_p_	Pore volume
VS	Volatile solids
WF–control	Vample of waste wafers
WF–Ch/P	Sample of waste wafers with carrier Ch/P
WF–Ch/P	Sample of waste wafers and waste cheese
WFC–Ch/P	Sample of waste wafers and waste cheese with carrier Ch/P
WF–Control 1, WF–Ch/P 1, WFC–Control 1, WFC–Ch/P 1	Samples collected from the first stage (1) of AD
WF–Control 2, WF–Ch/P 2, WFC–Control 2, WFC–Ch/P 2	Samples collected from the last stage (2) of AD

## References

[1] Wang T., Wang J., Jiazi Niu J., Guo P., Peng C., He R., Hui Z., Gao W., Zhang Q. (2024). Synchronous improvement of methane production and digestate dewaterability in sludge anaerobic digestion by nanobubble. Bioresour. Technol..

[2] Assis T.I., Gonçalves R.F. (2022). Valorization of food waste by anaerobic digestion: A bibliometric and systematic review focusing on optimization. J. Environ. Manag..

[3] Yadav M., Joshi C., Paritosh K., Thakur J., Pareek N., Masakapalli S.K., Vivekanand V. (2022). Reprint of Organic waste conversion through anaerobic digestion: A critical insight into the metabolic pathways and microbial interactions. Metab. Eng..

[4] Zhou M., Yang H., Zheng D., Pu X., Liu Y., Wang L., Zhang Y., Deng L. (2019). Methanogenic activity and microbial communities characteristics in dry and wet anaerobic digestion sludges from swine manure. Biochem. Eng. J..

[5] Liu Y., Zhu Y., Jia H., Yong X., Zhang L., Zhou J., Cao Z., Kruse A., Wei P. (2017). Effects of different biofilm carriers on biogas production during anaerobic digestion of corn straw. Bioresour. Technol..

[6] Jin H.J., Yao X.Y., Tang C.C., Zhou A.J., Liu W., Ren Y.X., Li Z., Wang A., He Z.W. (2024). Magnetite modified zeolite as an alternative additive to promote methane production from anaerobic digestion of waste activated sludge. Renew. Energy.

[7] Langxian S., Lintong Z., Xin Y., Maoyou Y., Jialin L., Minchun H., Xidan F., Lianhua L. (2024). Dual roles in interspecies electron transfer of carbon-based materials for accelerating anaerobic digestion of food waste. Biochem. Eng. J..

[8] Pilarska A.A., Pilarski K., Adamski M., Zaborowicz M., Dorota Cais-Sokolińska D., Wolna-Maruwka A., Niewiadomska A. (2022). Eco-friendly and effective diatomaceous earth/peat (DEP) microbial carriers in the anaerobic biodegradation of food waste products. Energies.

[9] Pilarska A.A., Wolna-Maruwka A., Niewiadomska A., Jarosław Grządziel J., Gałązka A., Paluch E., Borowiak K., Pilarski K. (2022). Quantitative and qualitative changes in the genetic diversity of bacterial communities in anaerobic bioreactors with the diatomaceous earth/peat cell carrier. Cells.

[10] Fenice M., Gorrasi S. (2021). Advances in chitin and chitosan science. Molecules.

[11] Stefanowska K., Woźniak M., Dobrucka R., Ratajczak I. (2023). Chitosan with natural additives as a potential food packaging. Materials.

[12] Maliki S., Sharma G., Kumar A., Moral-Zamorano M., Baselga J., Florian J., Stadler F.J., García-Peñas A. (2022). Chitosan as a Tool for Sustainable Development: A Mini Review. Polymers.

[13] Li B., Shi Y., Shan C., Zhou Q., Ibrahim M., Wang Y., Wu G., Li H., Xie G., Sun G. (2013). Effect of chitosan solution on the inhibition of Acidovoraxcitrulli causing bacterial fruit blotch of watermelon. J. Sci. Food Agric..

[14] Bonde S., Chandarana C., Prajapati P., Vashi V. (2024). A comprehensive review on recent progress in chitosan composite gels for biomedical uses. Int. J. Biol. Macromol..

[15] Olajire A.A., Bamigbade L.A. (2021). Green synthesis of chitosan-based iron@ silver nanocomposite as adsorbent for wastewater treatment. Water Res. Ind..

[16] Yin M., Chen H. (2022). Unveiling the dual faces of chitosan in anaerobic digestion of waste activated sludge. Bioresour. Technol..

[17] Nie W., Lin Y., Wu X., Wu S., Li X., Cheng J.J., Yang C. (2023). Chitosan-Fe_3_O_4_ composites enhance anaerobic digestion of liquor wastewater under acidic stress. Bioresour. Technol..

[18] Tetteh E.K., Amo-Duodu G., Rathilal S. (2022). Biogas production from wastewater: Comparing biostimulation impact of magnetised-chitosan and-titania chitosan. Mater. Today Proc..

[19] Yilmazer S., Ozdeniz M.B. (2005). The effect of moisture content on sound absorption of expanded perlite plates. Build. Environ..

[20] Ates A., Altintig E., Demirel H., Yilmaz M. (2017). Comparative study on adsorptive removal of Cu, Pb, Zn heavy metals by modified perlite composites. Desalin. Water Treat..

[21] Khoshraftar Z., Masoumi H., Ghaemi A. (2023). On the performance of perlite as a mineral adsorbent for heavy metals ions and dye removal from industrial wastewater: A review of the state of the art. Case Stud. Chem. Environ. Eng..

[22] Yan Y., Jia G., Zhang Y., Gao Y., Li Z. (2023). The influence of expanded perlite as a bio-carrier on the freeze-thaw properties of self-healing concreto. Constr. Build. Mater..

[23] Ivankovic T., Kontek M., Mihalic V., Ressler A., Jurisic V. (2022). Perlite as a biocarrier for augmentation of biogas-producing reactors from olive (*Olea europaea*) waste. Appl. Sci..

[24] Vijaya Y., Subbaiah M.V., Reddy A.S., Krishnaiah A. (2010). Equilibrium and kinetic studies of fluoride adsorption by chitosan coated perlite. Desalin. Water Treat..

[25] Demirçivi P. (2018). Synthesis and characterization of Zr(IV) doped immobilized cross-linked chitosan/perlite composite for acid orange II adsorption. Int. J. Biol. Macromol..

[26] Farrokhi Z., Sadjadi S., Raouf F., Bahri-Laleh N. (2022). Novel bio-based Pd/chitosan-perlite composite bead as an efficient catalyst for rapid decolorization of azo dye. Inorg. Chem. Comm..

[27] Pilarska A.A., Wolna-Maruwka A., Pilarski K., Janczak D., Przybył K., Gawrysiak-Witulska M. (2019). The use of lignin as a microbial carrier in the co-digestion of cheese and wafer waste. Polymers.

[28] Cabuk M. (2016). Electrorheological properties of biodegradable chitosan/expanded perlite composites. JOTCSA.

[29] (2006). Fermentation of Organic Materials Characterization of the Substrate, Sampling, Collection of Material Data, Fermentation Tests.

[30] ISO (1993). Guide to the Expression of Uncertainty in Measurement (GUM).

[31] (1985). Characterisation of the Substrate, Sampling, Collection of Material Data, Fermentation Tests.

[32] Pilarska A.A., Marzec-Grządziel A., Paluch E., Pilarski K., Wolna-Maruwka A., Kubiak A., Kałuża T., Kulupa T. (2023). Biofilm formation and genetic diversity of microbial communities in anaerobic batch reactor with polylactide (PLA) addition. Int. J. Mol. Sci..

[33] Team R.C. (2016). A Language and Environment for Statistical Computing.

[34] Callahan B.J., McMurdie P.J., Rosen M.J., Han A.W., Johnson A.J.A., Holmes S.P. (2016). DADA2: High-resolution sample inference from Illumina amplicon data. Nat. Methods.

[35] Wang Q., Cole J.R. (2024). Updated RDP taxonomy and RDP Classifier for more accurate taxonomic classification. Microbiol. Resour. Announc..

[36] Murali A., Bhargava A., Wright E.S. (2018). IDTAXA: A novel approach for accurate taxonomic classification of microbiome sequences. Microbiome.

[37] McMurdie P.J., Holmes S. (2013). cPhyloseq: An R package for reproducible interactive analysis and graphics of microbiome census data. PLoS ONE.

[38] Sweah Z.J., Malk F.H., Hussain W.A. (2020). Determination of the Optical Parameter from Chitosan Doping with Nicotine. AIP Conf. Proc..

[39] Hasan S., Ghosh T.K., Boddu V.M. (2008). Dispersion of *chitosan* on *perlite* for enhancement of copper(II) adsorption capacity. J. Hazard. Mater..

[40] Swayampakula K., Boddu V.M., Abburi K. (2009). Competitive adsorption of Cu (II), Co (II) and Ni (II) from their binary and tertiary aqueous solutions using chitosan–coated perlite beads as biosorbent. J. Hazard. Mater..

[41] Patil S.B., Sawant K.K. (2011). Chitosan microspheres as a delivery system for nasal insufflation. Colloids Surf. B Biointerfaces.

[42] Nguyen T.V., Nguyen T.T.H., Wang S.L., Vo T.P.K., Nguyen A.D. (2017). Preparation of chitosan nanoparticles by TPP ionic gelation combined with spray drying, and the antibacterial activity of chitosan nanoparticles and a chitosan nanoparticle–amoxicillin complex. Res. Chem. Intermed..

[43] Vaezifar S., Razavi S., Golozar M.A., Karbasi S., Morshed M., Kamali M. (2013). Effects of some parameters on particle size distribution of chitosan nanoparticles prepared by ionic gelation method. J. Clust. Sci..

[44] Dhawade P.P., Jagtap R.N. (2012). Characterization of the glass transition temperature of chitosan and its oligomers by temperature modulated differential scanning calorimetry. Adv. Appl. Sci. Res..

[45] Karaipekli A., Biçer A., Sarı A., Tyagi V.V. (2017). Thermal characteristics of expanded perlite/paraffin composite phase change material with enhanced thermal conductivity using carbon nanotubes. Energy Conv. Manag..

[46] Major N., Sørensen S.J.S.J., Ban D., Nesme J., Grosch R., Ban S.G., Schikora A., Cerne M., Schierstaedt J. (2022). Influence of sewage sludge stabilization method on microbial community and the abundance of antibiotic resistance genes. Waste Manag..

[47] Świątczak P., Cydzik-Kwiatkowska A., Rusanowska P. (2017). Microbiota of anaerobic digesters in a full-scale wastewater treatment plant. Arch. Environ. Prot..

[48] Deng P., Wang L., Li X., Zhang J., Jiang H. (2023). *Geobacter grbiciae*—A new electron donor in the formation of co-cultures via direct interspecies electron transfer. Microbiol. Res..

[49] Liu S., Liang D., Wang Y., He W., Feng Y. (2025). Impact of carrier capacitance on *Geobacter* enrichment and direct interspecies electron transfer under anaerobic conditions. Bioresour. Technol..

[50] Yan Y., Zhang J., Tian L., Yan X., Du L., Leininger A., Zhang M., Li N., Ren Z.J., Wang X. (2023). DIET-like mutualism of *Geobacter* and methanogens at specific electrode potential boosts production of both methane and hydrogen from propionate. Water Res..

[51] Hu P., Xiao M., Wang N., Zhang S., Shi J., Shi J., Tang T., Liu L. (2024). Metagenome reveals the possible mechanism that microbial strains promote methanogenesis during anaerobic digestion of food waste. Environ. Res..

[52] Matheri A.N., Eloko N.S., Ntuli F., Ngila J.C. (2020). Influence of pyrolyzed sludge use as an adsorbent in removal of selected trace metals from wastewater treatment. Case Stud. Chem. Environ. Eng..

[53] Yang H., Yan R., Chen H., Lee D.H., Zheng C. (2007). Characteristics of hemicellulose, cellulose and lignin pyrolysis. Fuel.

[54] Hantoko D., Kanchanatip A.E., Yan M., Weng Z., Gao Z., Zhong Y. (2019). Assessment of sewage sludge gasification in supercritical water for H_2_-rich syngas production. Process Saf. Environ. Prot..

[55] Mathaba M., Daramola M.O. (2020). Effect of chitosan’s degree of deacetylation on the performance of PES membrane infused with chitosan during AMD treatment. Membranes.

[56] Sikorski D., Gzyra-Jagieła K., Draczyński Z. (2021). The kinetics of chitosan degradation in organic acid solutions. Mar. Drugs.

[57] Mohammadnezhad J., Khodabakhshi-Soreshjani F., Bakhshi H. (2016). Preparation and evaluation of chitosan-coated eggshell particles as copper(II) biosorbent. Desalin. Water Treat..

[58] Dandil S., Sahbaz D.A., Acikgoz C. (2019). Adsorption of Cu(II) ions onto crosslinked chitosan/Waste Active Sludge Char (WASC) beads: Kinetic, equilibrium, and thermodynamic study. Int. J. Biol. Macromol..

[59] Jiang L., Jia G., Jianga C., Li Z. (2020). Sugar-coated expanded perlite as a bacterial carrier for crack-healing concrete applications. Constr. Build. Mater..

[60] Yan Y., Liu W., Li Z., Jia G., Zhang Y., Ma G., Gao Y. (2024). Mechanical properties and frost resistance of self-healing concrete based on expanded perlite with different particle sizes as microbial carrier. Constr. Build. Mater..

[61] Sahbaza D.A., Acikgoz C. (2017). Adsorption of a textile dye Ostazin Black NH from aqueous solution onto chitosan-coated perlite beads. Desalin. Water Treat..

[62] Zamrisham N.A.F., Idrus S., Harun M.R., Ab Razak M.S., Jaman K. (2024). Biogas production by integrating lava rock, red clay & ceramic bio ring as support carrier in treatment of landfill leachate with liquidised food waste. Biochem. Eng. J..

