# Supplementary material for: The Use of Chitosan/Perlite Material for Microbial Support in Anaerobic Digestion of Food Waste
Source: Materials (Basel). 2025 Jul 26;18(15):3504. doi: 10.3390/ma18153504 (PMC12347546; doi:10.3390/ma18153504)
Supplement: Supplementary file 1 [file materials-18-03504-s001.zip › materials-3178080-supplementary.pdf]

materials

Supplementary Materials

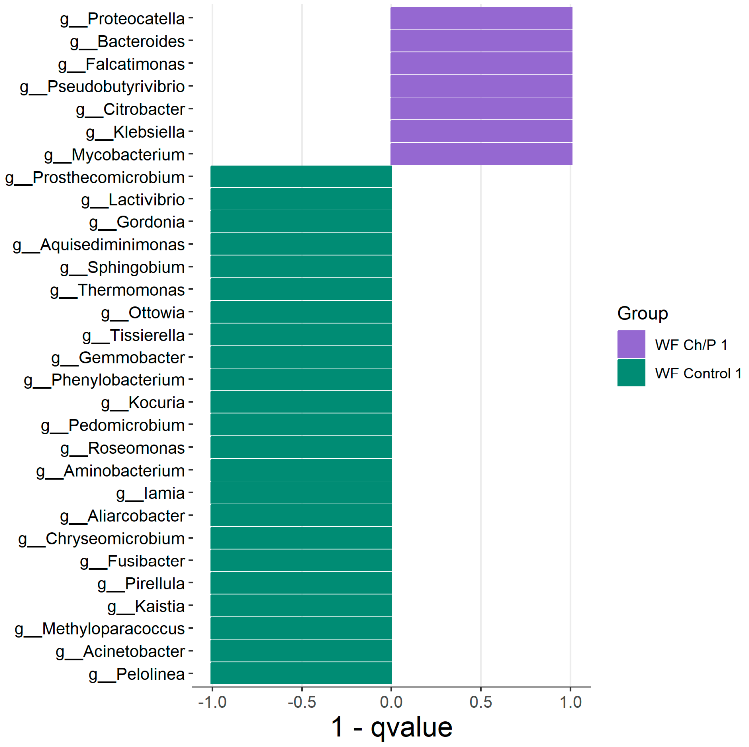

Figure S1: Comparison (MetaStat analysis) of bacterial genus composition between WF–Control 1 and WF–Ch/P 1.

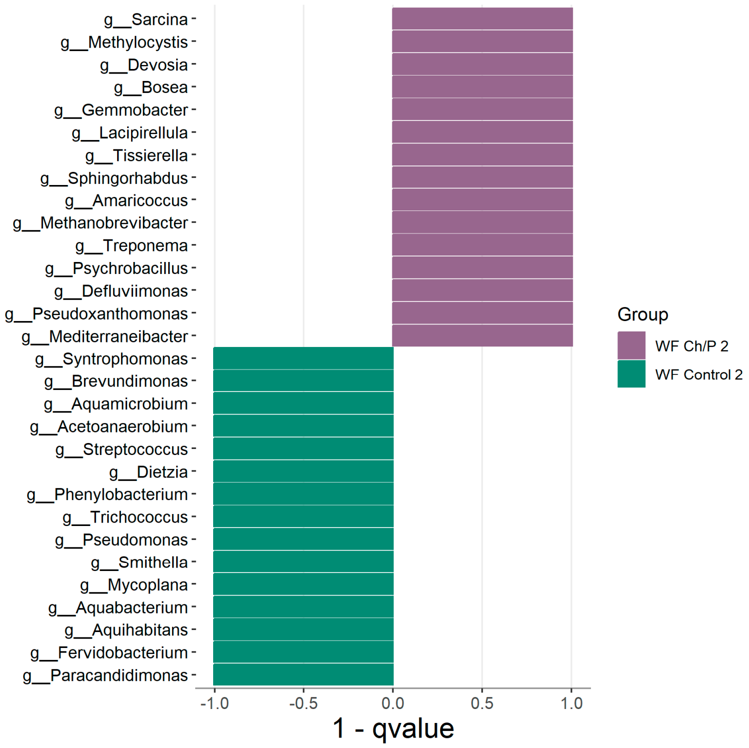

Figure S2: Comparison (MataStat analysis) of bacterial genus composition between WF–Control 2 and WF–Ch/P 2.

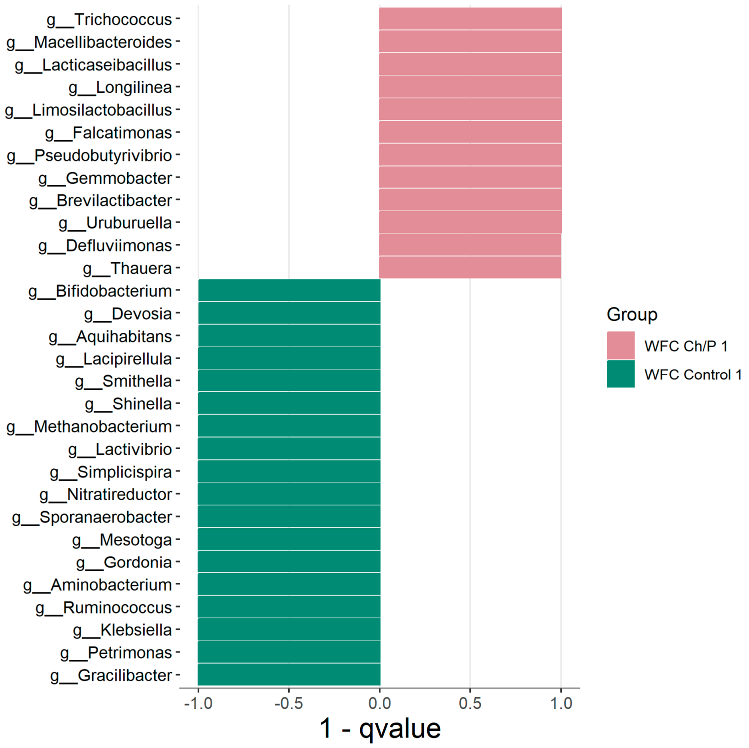

Figure S3: Comparison (MetaStat analysis) of bacterial genus composition between WFC– Control 1 and WFC–Ch/P 1.

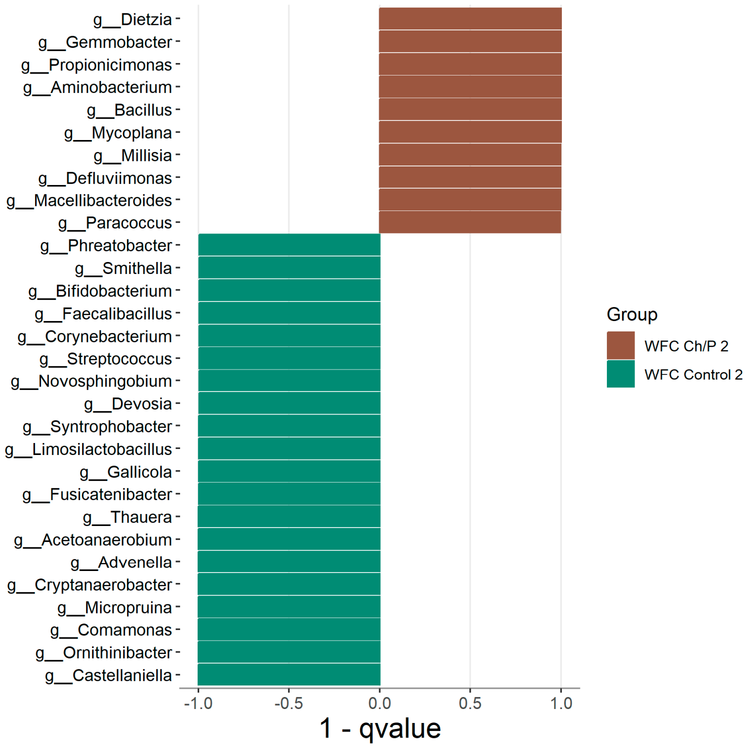

Figure S4: Comparison (MetaStat analysis) of bacterial genus composition between WFC– Control 2 and WFC–Ch/P 2.

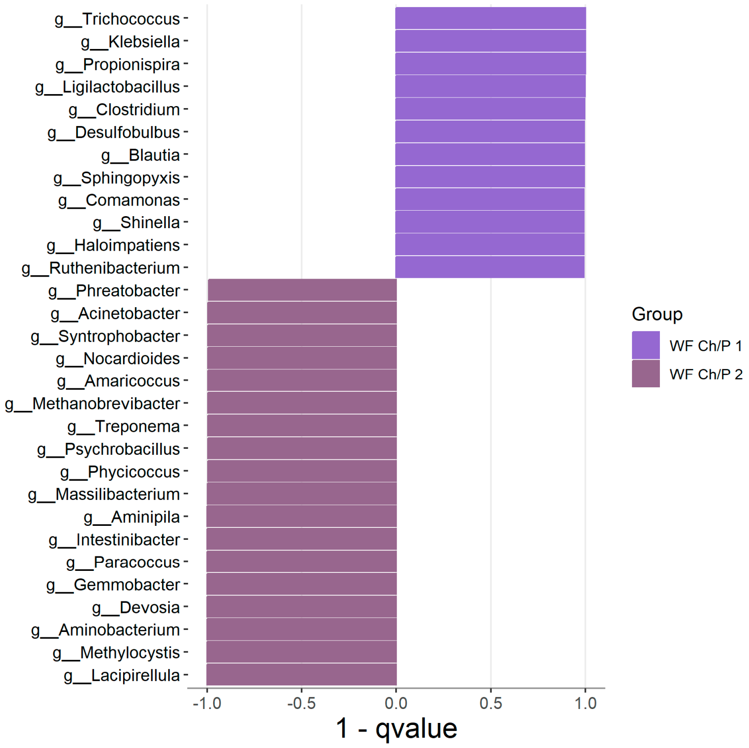

Figure S5: Comparison (MetaStat analysis) of bacterial genus composition between WF–Ch/P 1 and WF–Ch/P 2.

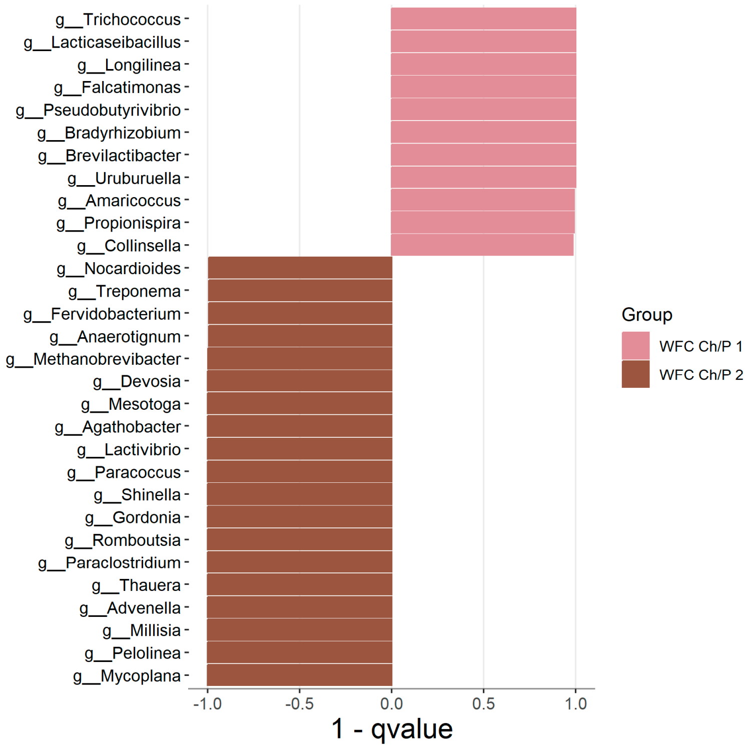

Figure S6: Comparison (MetaStat analysis) of bacterial genus composition between WFC–Ch/P 1 and WFC–Ch/P 2.

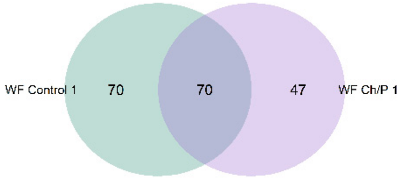

(**a**)

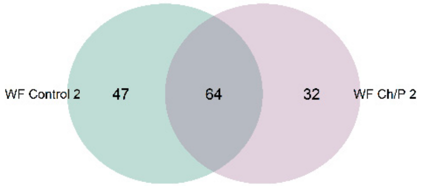

(**b**)

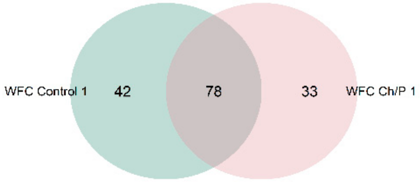

(**c**)

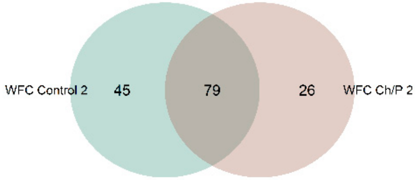

(**d**)

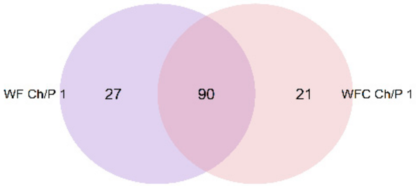

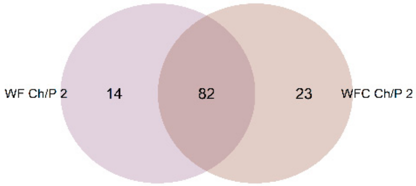

(e) **(f)**

Figure S7: Venn diagrams prepared based on the sequencing of the 16S rRNA gene. The data shown in the diagram refer to site–specific unique ASVs and the core microbiome (a–f).
